# Force‐transmitting structures in the digital pads of the tree frog *Hyla cinerea*: a functional interpretation

**DOI:** 10.1111/joa.12860

**Published:** 2018-08-19

**Authors:** Julian K. A. Langowski, Henk Schipper, Anne Blij, Frank T. van den Berg, Sander W. S. Gussekloo, Johan L. van Leeuwen

**Affiliations:** ^1^ Experimental Zoology Group Wageningen University & Research Wageningen The Netherlands

**Keywords:** attachment organ, bioadhesion, collagen, connective tissue, fibre‐matrix‐composite, material stiffness, shear load, smooth muscle

## Abstract

The morphology of the digital pads of tree frogs is adapted towards attachment, allowing these animals to attach to various substrates and to explore their arboreal habitat. Previous descriptions and functional interpretations of the pad morphology mostly focussed on the surface of the ventral epidermis, and little is known about the internal pad morphology and its functional relevance in attachment. In this study, we combine histology and synchrotron micro‐computer‐tomography to obtain a comprehensive 3‐D morphological characterisation of the digital pads (in particular of the internal structures involved in the transmission of attachment forces from the ventral pad surface towards the phalanges) of the tree frog *Hyla cinerea*. A collagenous septum runs from the distal tip of the distal phalanx to the ventral cutis and compartmentalises the subcutaneous pad volume into a distal lymph space and a proximal space, which contains mucus glands opening via long ducts to the ventral pad surface. A collagen layer connects the ventral basement membrane via interphalangeal ligaments with the middle phalanx. The collagen fibres forming this layer curve around the transverse pad‐axis and form laterally separated ridges below the gland space. The topological optimisation of a shear‐loaded pad model using finite element analysis (FEA) shows that the curved collagen fibres are oriented along the trajectories of the maximum principal stresses, and the optimisation also results in ridge‐formation, suggesting that the collagen layer is adapted towards a high stiffness during shear loading. We also show that the collagen layer is strong, with an estimated tensile strength of 2.0–6.5 N. Together with longitudinally skewed tonofibrils in the superficial epidermis, these features support our hypothesis that the digital pads of tree frogs are primarily adapted towards the generation and transmission of friction rather than adhesion forces. Moreover, we generate (based on a simplified FEA model and predictions from analytical models) the hypothesis that dorsodistal pulling on the collagen septum facilitates proximal peeling of the pad and that the septum is an adaptation towards detachment rather than attachment. Lastly, by using immunohistochemistry, we (re‐)discovered bundles of smooth muscle fibres in the digital pads of tree frogs. We hypothesise that these fibres allow the control of (i) contact stresses at the pad–substrate interface and peeling, (ii) mucus secretion, (iii) shock‐absorbing properties of the pad, and (iv) the macroscopic contact geometry of the ventral pad surface. Further work is needed to conclude on the role of the muscular structures in tree frog attachment. Overall, our study contributes to the functional understanding of tree frog attachment, hence offering novel perspectives on the ecology, phylogeny and evolution of anurans, as well as the design of tree‐frog‐inspired adhesives for technological applications.

## Introduction

Tree frogs possess adhesive digital pads that enable these animals to climb vertical substrates, hence allowing the exploration of arboreal habitats, the evasion of ground‐borne predators and the disclosure of otherwise unreachable food sources. Studying the morphology and functioning of these organs helps to unravel the ecology (Green & Simon, 1986; Emerson, 1991), evolution (Moen et al. 2013; Sustaita et al. 2013) and phylogeny (Green, 1979, 1980; McAllister & Channing, 1983; Hertwig & Sinsch, 1995) of tree frogs, as well as to design bioinspired adhesives (Murarash et al. 2011; Drotlef et al. 2013; Tsipenyuk & Varenberg, 2014; Iturri et al. 2015; Zhang et al. 2016; Xue et al. 2017).

**Figure 1:**
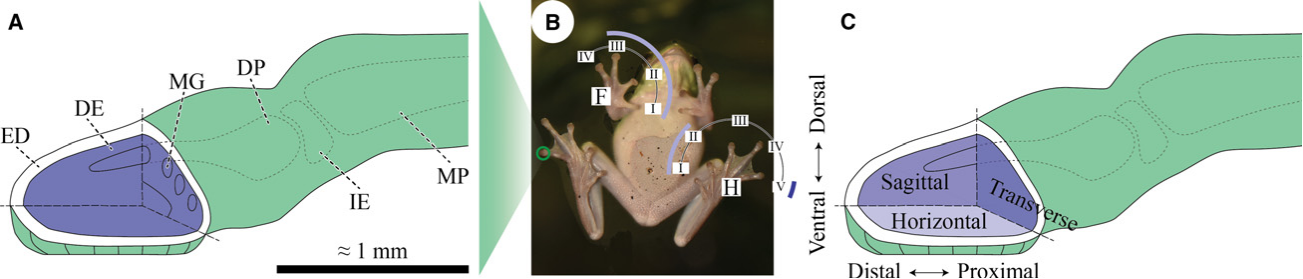
(A) Basic morphological terminology of the digital tip and the distal interphalangeal joint of the tree frog *Hyla cinerea*. The blue region depicts the largely uncharacterised dermal and subdermal space including connective, muscular, vascular and other tissues. (B) Nomenclature and usage of the digits I–IV in the right forelimb (F) and I–V in the left hindlimb (H) [light blue arcs (F_I–III_, H_I–II_): histology/immunohistochemistry, dark blue arc (H_V_): μ‐CT]. (C) Definition of terms of anatomical location and of the cutting and viewing planes. DE, dermis; DP, distal phalanx; ED, epidermis; IE, intercalary element; MG, mucus gland; MP, middle phalanx.

The digital pads are macroscopically smooth, soft (Scholz et al. 2009), and proposedly adhere by wet adhesion (v. Wittich, 1854; Schuberg, 1891; Siedlecki, 1909; Nachtigall, 1974; Emerson & Diehl, 1980; Hanna & Barnes, 1991): tree frogs secrete a watery mucus into the gap between pad and substrate (Blackwall, 1845; Federle et al. 2006), which may cause surface‐tension‐dependent capillary forces and viscosity‐dependent hydrodynamic forces (Barnes, 2012; Endlein & Barnes, 2015). The surface of the adhesive epidermis on the ventral (throughout this paper, we use ‘ventral’ to describe the palmar/plantar side of the digits; Fig. 1) side of the distal digital segment (for the digital segments and the phalanges, we use ‘distal’ and ‘middle’ to describe the ultimate/terminal and penultimate/subterminal ones, respectively; Fig. 1A) consists of prismatic cells separated by channels and covered with nanoscopic cellular protrusions, so‐called ‘nanopillars’ (Scholz et al. 2009), forming a hierarchical micro‐ to nanoscopic surface pattern (Ernst, 1973a). This epidermal morphology presumably evolved convergently in multiple tree frog clades (Green, 1979; McAllister & Channing, 1983; Lee et al. 2001; Barnes et al. 2013) and could facilitate (in addition to wet adhesion) attachment mechanisms such as mechanical interlocking (Emerson & Diehl, 1980), suction (Mohnike, 1879) and van der Waals interactions (Emerson & Diehl, 1980; Federle et al. 2006).

Previously, the discussion on the generation of adhesion and friction (i.e. the attachment force normal and parallel to the substrate surface, respectively) in tree frogs strongly focused on the superficial epidermis (Ernst, 1973a; Hanna & Barnes, 1991). The morphology and attachment‐related functions of dermal and subcutaneous structures of the digital tip, such as the transmission of adhesion and friction forces from the epidermis to the skeleton and hence to the rest of the body, remained largely unstudied. Here, we define the digital tip as the distal phalanx and all subdermal, dermal and epidermal tissues (Ernst, 1973a,b; Hertwig & Sinsch, 1995) surrounding it. Dermal and subdermal structures include connective (mainly collagenous) tissue, muscle fibres, blood vessels, a lymph space and mucus glands (Fig. 1A; Siedlecki, 1910; Ernst, 1973a; Nakano & Saino, 2016). To our knowledge, the adhesive epidermis is mechanically linked to the distal phalanx and the rest of the body by connective tissue only. Various authors described different collagenous structures, such as loose connective tissue forming the basement membrane and the dermal stratum spongiosum (Leydig, 1868; Schuberg, 1891; Siedlecki, 1910; Noble & Jaeckle, 1928; Nakano & Saino, 2016), collagen fibres traversing the distal lymph space (v. Wittich, 1854; Leydig, 1868; Dewitz, 1883), and laterally separated bundles of collagenous fibres running along the ventral pad surface (Noble & Jaeckle, 1928). However, others termed the fibres in the lymph space (Schuberg, 1891) and the ventral bundles (Siedlecki, 1910) muscular instead of collagenous structures, whereas more recently the presence of muscular structures was negated completely (except for smooth muscle cells surrounding the mucus glands; Ernst, 1973b; Mizuhira, 2004). Furthermore, the 3‐D arrangement of connective (and muscular) tissues, lymph space and skeleton (and accordingly the potential of these structures for force transmission) is largely unknown.

In the light of a recent report of a load of up to 14.4 times the body weight withstood by a single digital pad during landing in *Trachycephalus resinifictrix* (Bijma et al. 2016), we address in this paper the morphology and function, in particular in the transmission of attachment forces, of dermal and subcutaneous structures in the digital pads of the tree frog *Hyla cinerea*. As tree frogs arguably encounter mostly vertical substrates while climbing up and down in their arboreal habitat, we expect that the pads are primarily adapted towards the transmission of friction forces (i.e. shear loads). We employ a combination of histology, immunohistochemistry and synchrotron micro‐computer‐tomography (μ‐CT) to obtain a 3‐D characterisation of the pad morphology. Furthermore, we numerically predict the stiffness‐optimised topology of the collagenous tissue in the ventral pad region during shear loading, and we estimate (by measuring cross‐sectional areas) the tensile strength of the structures involved in force transmission. In combination, these approaches enable us to address the following questions.

1. How are force‐transmitting structures such as connective tissue, lymph space and skeletal elements distributed within the digital tip (and relative to each other)? Which pathways of transmission of adhesive and frictional forces do these structures accommodate?
2. How do the pad and adjacent structures transmit shear loads equivalent to several times the body weight?
3. Are there muscular structures involved in force transmission? If so, what function(s) could these structures fulfil?

## Materials and methods

### Ethical statement

All animals used in this study were bought from legal vendors and were not killed for the purpose of this research. Therefore, this research is not considered as an animal experiment by the animal ethics committee of Wageningen University & Research (WUR).

### Experimental animals

For morphological analyses, we used three adult *Hyla cinerea* that died of unknown causes (post mortem snout‐vent‐length 40–46 mm, body mass 6.2–8.2 g, age ≥ 1 year). We collected the distal limbs at most 5:30 h after death by disarticulation of the elbow and knee joints. Until further use, the right forelimb (F) and the left hindlimb (H; Fig. 1B) of each individual were fixed and decalcified for 2–12 weeks in Bouin's liquid [37% formaldehyde, saturated picric acid and acetic acid (Merck, USA) with a ratio of 5:15:1] and subsequently stored in 70% ethanol, which was renewed multiple times. All the following steps were executed at room temperature, unless mentioned otherwise.

### Histology

Before histological staining, the two most distal segments of digits F_I_, F_II_ and F_III_ of the forelimb, and of digits H_I_ and H_II_ of the hindlimb (Fig. 1B) were cut through the central part of the middle phalanx. For easy handling and correct alignment of the samples with respect to the desired cutting planes, they were pre‐embedded in small blocks of agarose gel [1% low‐melting agarose (Sigma‐Aldrich, USA) in demineralised water] before dehydrating and embedding the agarose blocks in paraffin (KP Paraclean I, VWR International B.V., The Netherlands; see Appendix S4). The embedded samples were cut into 5 μm thick sections using a microtome (Microm 305S, Microm GmbH, Germany) and placed on egg‐glycerin‐coated object slides (Menzel, Germany). Digit F_I_ was cut parallel to the ventral pad surface (i.e. horizontal sections), F_II_ parallel to the median digital plane (i.e. sagittal sections), and F_III_ perpendicularly to the longitudinal digital axis (i.e. transverse sections; Fig. 1C). Digits H_I_ and H_II_ were cut sagitally and transversally, respectively.

The samples on every second object slide in sequence (transverse and horizontal sections) and on the slides holding the left half of the digits (sagittal sections), respectively, were stained using Crossmon's light green trichrome including Mayer's haematoxylin and Alcian blue (see Appendix S4). Images of the stained sections were obtained using a digital microscope camera (DFC450c, Leica, Germany) mounted on an upright microscope (DM6b, Leica) with a HC PL APO 40×/0.85 objective controlled with the Leica Application Suite x (Version 2.0). High‐resolution images of the whole sections were obtained by merging tile‐scanned images. Post‐processing (cropping, rotating, scaling, white balancing, and arranging) of the images was done in Photoshop CC (Version 2017.1.1, Adobe Systems, USA) and in Illustrator CS6 (Version 16.0.3, Adobe). Geometrical parameters of interest were measured with ImageJ (Version 1.51f, National Institutes of Health, USA).

### Immunohistochemistry

To revise the presence of muscular tissues within the digital tip, we stained the remaining slides of frogs 1 and 2 using an actin‐antibody (A5228, Merck), which is specific for smooth‐muscle‐α‐actin (Skalli et al. 1986). Before immunohistochemical staining (see Appendix S5), the sections were deparaffinised towards demineralised water, endogenous peroxidase was removed with a solution of 0.3% H_2_O_2_ (Merck) in buffer, and aspecific antibody‐binding was blocked by the application of 10% goat serum (Vector Laboratories, USA) in combination with 1% acetylated bovine serum albumin (BSA‐c; Aurion, Netherlands). The primary α‐actin‐antibody was applied overnight at 4 °C (dilution 1:400), followed by a secondary goat‐anti‐mouse/horseradish‐peroxidase‐antibody (Agilent, Canada) for 45 min (dilution 1:100). Structures containing smooth‐muscle‐α‐actin were stained brown using 3,3’‐diaminobenzidine (7–10 min; Sigma) as substrate. Finally, we applied Mayer's haematoxylin as described above as counterstain, and dehydrated and sealed the sections with DPX mounting medium (VWR, USA). Imaging and post‐processing of the images were done as described above.

### Synchrotron micro‐computer‐tomography

The digit used for μ‐CT (Frog 3, H_V_; Fig. 1B) was dissected, fixed and stored as described for histology. The digit was transferred into phosphotungstic acid (PTA; 0.3% PTA in 70% ethanol; according to Metscher, 2009) contrast stain to enhance the contrast of the soft tissues of interest. The PTA‐stain was refreshed several times over the course of 9 days to ensure penetration of the whole sample. Before scanning, the sample was washed in 70% ethanol and dehydrated stepwise (45 min 80% ethanol, 30 min 90% ethanol, 20 min 96% ethanol, 2 × 15 min 100% ethanol); 100% ethanol was used as scanning medium.

The μ‐CT scan of the digit was acquired at the Tomographic Microscopy and Coherent Radiology beamline (TOMCAT, X02DA) of the Swiss Light Source facility at the Paul Scherrer Institute, Switzerland, using a monochromatic 14‐keV‐beam with almost parallel geometry and a LuAG:Ce‐scintillator. A high‐quality optical microscope (Optique Peter, France) with an UPLAPO10x‐objective (Olympus, Japan; numerical aperture 0.4) was used in combination with a pco.edge 5.5 camera (PCO AG, Germany; exposure time 100 ms, 2560 × 2160 pixels, pixel size 6.5 × 6.5 μm^2^), resulting in an effective pixel size of 0.65 × 0.65 μm^2^ and a field of view of 1.7 × 1.4 mm^2^. We mounted a pipette tip containing the sample within the scanning liquid on a rotation platform (ABRT150, Aerotech, USA) and rotated the sample around its approximate longitudinal axis, which was aligned perpendicular to the beam. The scan angle was varied from 0° to 180° in 0.1°‐steps. The scans were reconstructed using propagation‐based phase contrast imaging as described by Paganin et al. (2002). Because the sample was larger than the field of view, it was scanned in two batches along the proximal‐distal digit‐axis (2160 images per batch) with an overlap of 262 images (i.e. 170 μm). The batches were merged and overlapping images were removed.

Filtering of the reconstructed transverse sections and segmentation of the structures of interest were done in Seg3D (Version 2.4.0, NIH, USA). To reduce the efforts of computation and manual segmentation, every eighth transverse section was imported and the scanned volume was cropped closely around the sample; this resulted in 1759 × 1754 × 410 voxels (each with a size of 0.65 × 0.65 × 5.2 μm^3^) in transversal, dorsoventral and proximal‐distal direction, respectively. We used grey‐value thresholding on median filtered sections to mask most of the background voxels, which were then removed from the original sections, and automatic histogram equalisation to enhance the contrast of the internal digital structures (see Appendix S3). The phalanges, intercalary element, tendons, ligaments, ventral collagen, smooth muscle fibres and mucus glands were segmented section‐wise by masking the according structures manually using the ‘polyline’ and ‘paintbrush’ tools. To distinguish the different tissues, we considered density differences (e.g. muscle fibres appeared brighter than collagenous fibres), differences in fibre orientation (to distinguish individual collagen bands), and knowledge on the digital morphology from the histological sections and from literature. The outlines of most structures were clearly distinguishable. However, the segmentation of fine structures (e.g. the side arms of the ligaments, the ventral collagen ridges, and the mucus ducts within the ventral epidermis) and of connections between collagenous and other tissues (i.e. entheses and apophyses) was less accurate due to the absence of distinct visual features.

### Topological optimisation of a shear‐loaded pad model

To provide a functional explanation for the distribution of collagenous tissue in the digital pad, we performed a topological optimisation of a shear‐loaded pad model via finite element analysis (FEA). This is the optimisation of the distribution of material within a design space for a set of boundary conditions and optimisation goals (Suresh, 2013). We created a simplified, non‐optimised geometrical model of the ventral cutis with the approximate dimensions of a real pad (0.45 mm high, 1 mm wide, 1.5 mm long; Fig. 10A) in Solidworks (Version 2015 SP5.0 Education Edition, Dassault Systèmes, USA). Proximally, material was recessed to model the neighbouring proximal epiphysis of the distal phalanx. We implemented three longitudinal rows of each five vertical holes (Ø = 0.1 mm, hole spacing = 0.14 mm, row spacing = 0.25 mm) to represent mucus ducts piercing the connective tissue. In contrast to the effective elastic modulus of the pad epidermis (Scholz et al. 2009; Barnes et al. 2011, 2013; Kappl et al. 2016), the elastic modulus *E*, yield strength σ and Poisson's ratio ν of deeper lying structures to our knowledge are unknown. We verified the independency of the qualitative optimisation results from variations in elastic modulus, and applied *E *= σ = 20 MPa and ν = 0.33, which is within the range of values reported for collagenous tissues (Biewener, 2008).

Using Paretoworks (Version 2017.04, SciArt, USA), we created a mesh of about 250 000 elements (edge length ≈ 13 μm). Displacement and removal of the ventral model surface were prohibited, and a normal tensile load of 3.815 mN (= 7 g × 9.81 m s^−2^ / 18, equivalent to an uniform distribution of the approximate body weight over all 18 digits) was applied on the most proximal model surface (red area in Fig. 10A_I_) to simulate a shear load as experienced by an animal attaching to a vertical surface with its head facing upwards. Using the geometrical lateral symmetry to reduce computational efforts, the initial model volume was reduced by 60% in 2.5%‐steps (see Appendix S7) while maintaining maximum stiffness. We exported the optimised geometry as .stl‐file (‘very fine’ resolution) and reimported it using Power Surfacing (Version 4.1.0008, IntegrityWare, USA) into Solidworks for post‐processing. The surface of the optimised geometry was remeshed with the ‘quad wrap’ function (polygon size = 2.2% of the length of the whole geometry); small mesh inaccuracies were corrected manually. The resulting surface mesh was transformed into a ‘medium’‐quality volume mesh. Using the in‐house FEA‐solver of Solidworks, we remeshed the non‐optimised and optimised model (with 365 000 and 187 000 elements, respectively; edge length ≈ 20 μm) and computed von Mises stresses to identify regions of low mechanical loading, as well as maximum principal stresses to visualise the trajectories of force transmission through the pad model. The same material properties and loading were used as described above.

## Results

**Figure 2:**
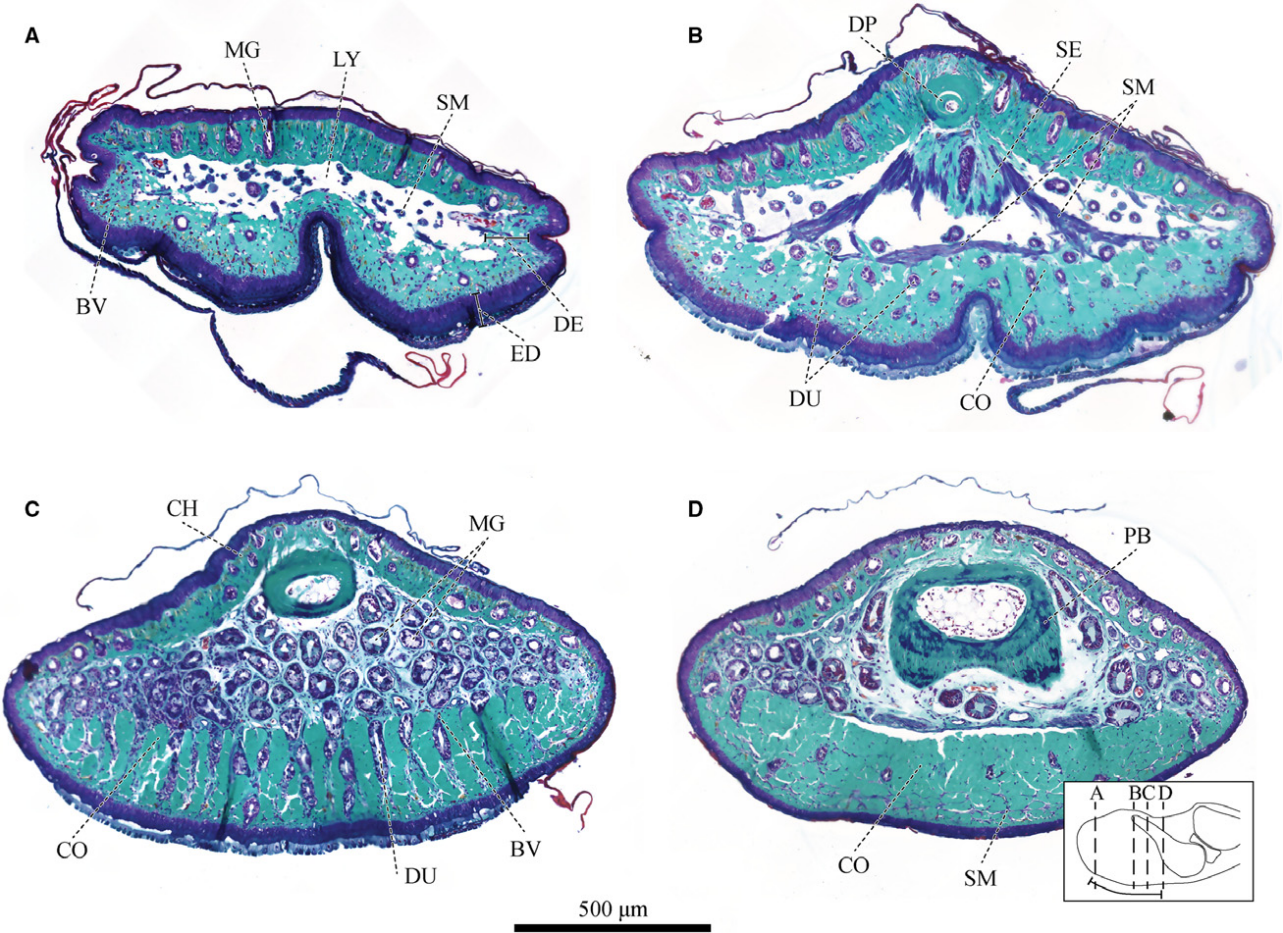
Series of transverse sections of a digital pad of *Hyla cinerea* (Frog 3, digit F_III_) from distal to proximal stained with Crossmon's light green trichrome including Mayer's haematoxylin and Alcian blue [see inset for the approximate locations and the extent of the adhesive ventral epidermis (curved solid line)] through (A) the lymph space, (B) the approximate septum plane, (C) the gland space and (D) the distal end of the base of the distal phalanx. BV, blood vessel; CH, chromatophore; CO, collagen tissue; DE, dermis; DP, distal phalanx; DU, mucus duct; ED, epidermis; LY, lymph space; MG, mucus gland; PB, base of the distal phalanx; SE, septum; SM, smooth muscle.

**Figure 3:**
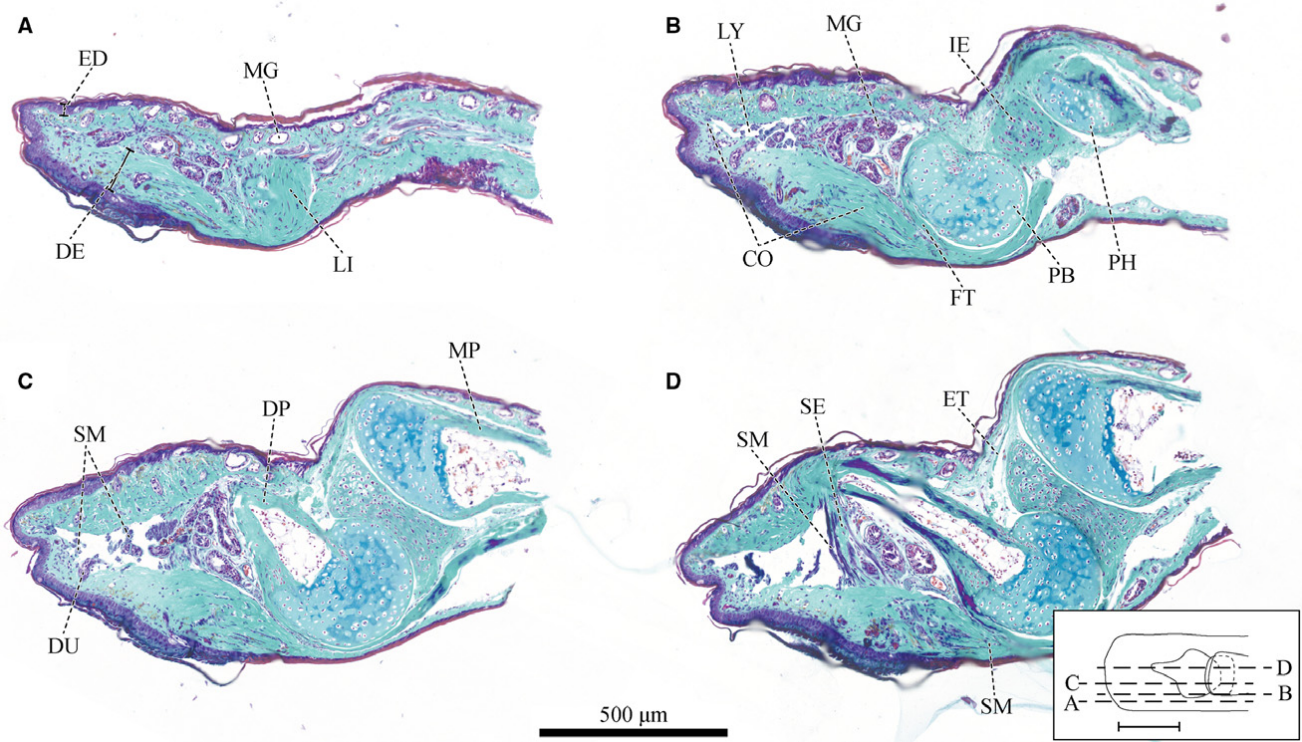
Series of sagittal sections of a digital pad of *Hyla cinerea* (Frog 3, digit H_I_) from lateral to mid‐sagittal stained as in Fig. 2 [see inset for the approximate locations and the extent of the adhesive ventral epidermis (solid line)] (A) close to the lateral epidermis, (B) through the lateral part of the base of the distal phalanx, (C) through the lateral part of the diaphysis of the distal phalanx, and (D) in an approximately mid‐sagittal plane. Abbreviations as in Fig. 2, with the following additions: ET, tendon of the extensor muscle; FT, tendon of the flexor muscle; IE, intercalary element; LI, ligament; MP, middle phalanx; PH, head of the middle phalanx.

**Figure 4:**
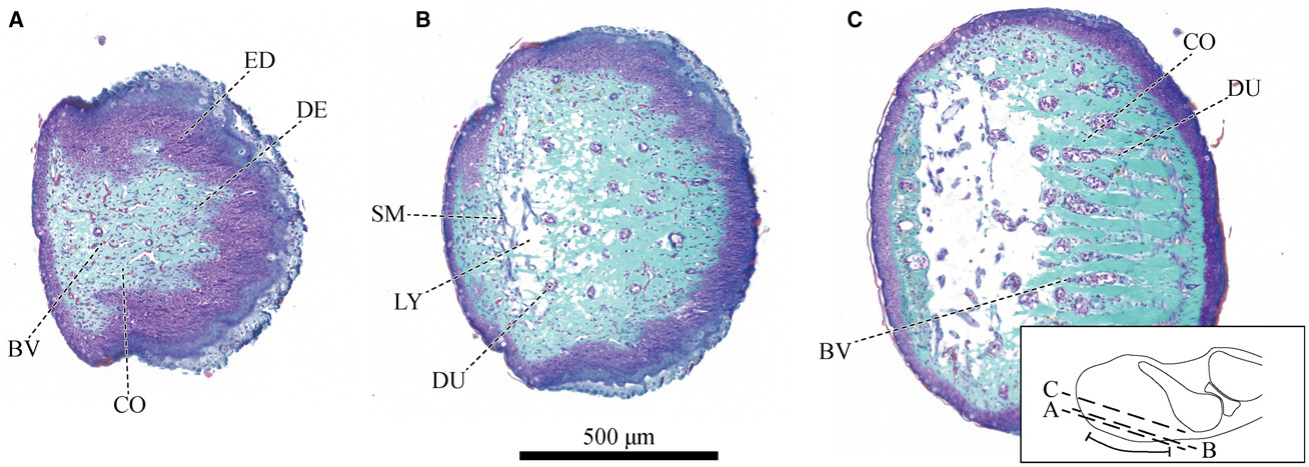
Series of horizontal sections of a digital pad of *Hyla cinerea* (Frog 3, digit F_I_) from ventral to dorsal stained as in Fig. 2 [see inset for the approximate locations and extent of the adhesive ventral epidermis (curved solid line)] through (A) the apical dermis (stratum spongiosum), (B) the ventral part of the ventral collagen layer, and (C) the dorsal part of the ventral collagen layer. Abbreviations as in Figs 2, 3.

**Figure 5:**
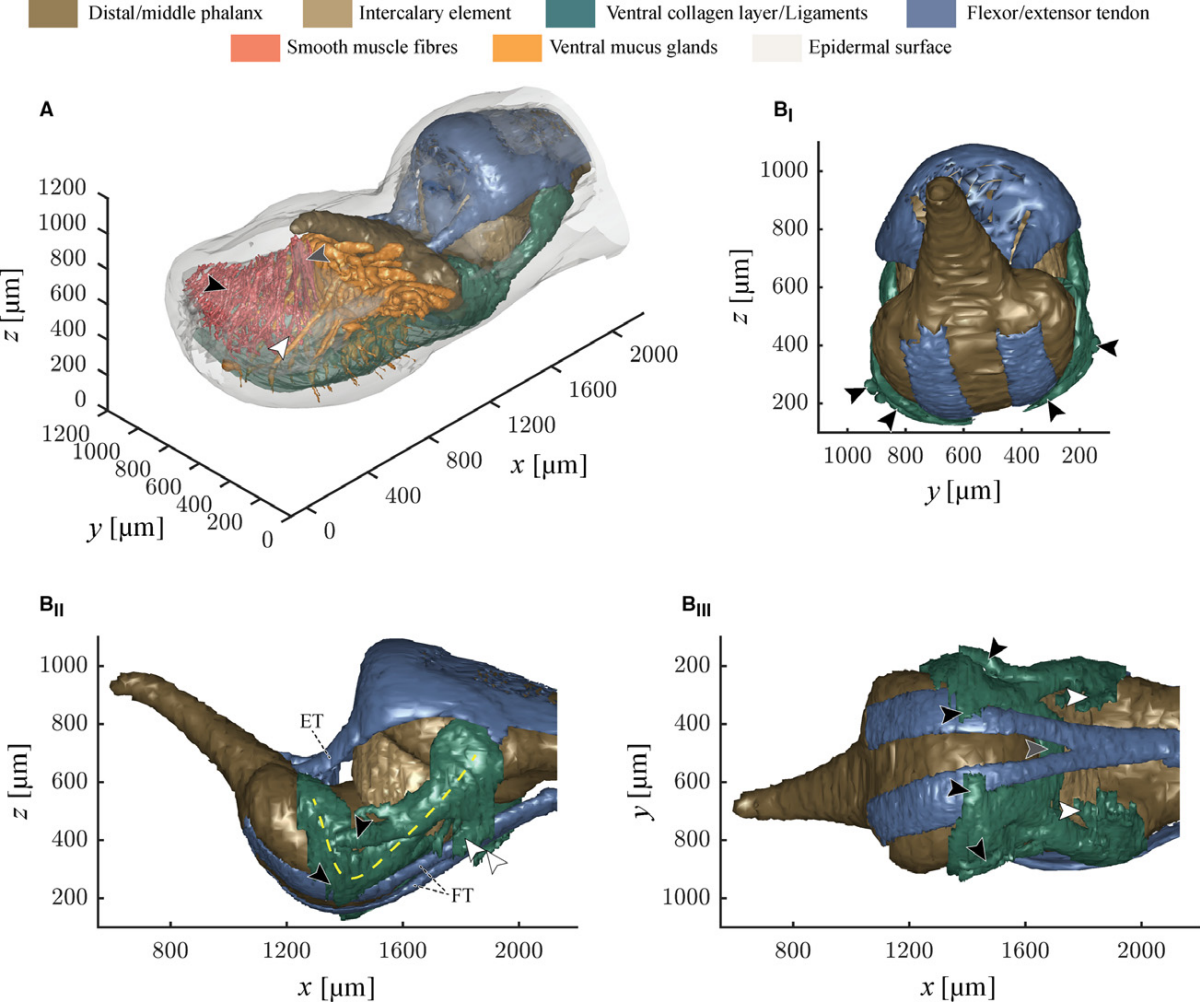
3‐D visualisation of the structures of force transmission in a digital pad of *Hyla cinerea* (Frog 3, digit H_V_). (A) 3‐D view of the whole digital tip and internal structures. Only half of the approximately bi‐laterally symmetric smooth muscle fibres are shown (black arrowhead: thin muscle fibre bundle; grey arrowhead: thick muscle fibre bundle; white arrowhead: distal‐cross‐lateral muscle fibre). (B_I_) Frontal, (B_II_) lateral and (B_III_) ventral view of the joint region: the collateral ligaments (yellow dashed line) give rise to several side arms, which connect to the ventral cutis in the middle digital segment (white arrowheads) and the ventral collagen layer (black arrowheads). The two collateral ligaments are connected via a medial strand that also connects to the intercalary element (grey arrowhead). Abbreviations as in Figs 2, 3, 4. *x*, longitudinal spatial coordinate; *y*, lateral spatial coordinate; *z*, vertical spatial coordinate.

The general bauplan of the digital tip of *Hyla cinerea* does not vary in between digits, limbs and individuals (see Appendix S6), and is described below. Epidermal tissue encloses the digital tip (Figs 2, 3, 4 and 5A). Collagenous tissue and blood vessels form the dermis. The dorsal dermis contains numerous mucus glands, which are not found in the ventral dermis. The nearly hemispherical proximal epiphysis of the distal phalanx (referred to as the base of the distal phalanx) fills the ventroproximal part of the subdermal pad space. Distally, the distal phalanx tapers into a curved diaphysis protruding approximately halfway into the digital tip, with an upward pointing angle of about 30–40°. A collagenous septum runs from the distal tip of the distal phalanx towards the ventral cutis and compartmentalises the subdermal space: lymph fills a substantial portion of the space distal to the septum (referred to as lymph space; Noble & Jaeckle, 1928; Nakano & Saino, 2016), and a dense cluster of glands occupies the space proximal to the septum (referred to as gland space). Mucus ducts connecting the glands to the ventral pad surface and numerous bundles of smooth muscle fibres traverse the lymph space. Ventrally, a layer of collagen fibres runs longitudinally through the digital pad (referred to as ventral collagen layer). As mentioned before, we will focus on the structures relevant to force transmission, both within the distal interphalangeal joint region and in the digital tip.

### Phalanges and the distal interphalangeal joint

A biconcave intercalary element, which is wider than thick, is found between the distal and middle phalanx (Fig. 3D). The articulating surface of the intercalary element with the distal phalanx is concave and counterfits the base of the distal phalanx, whereas the articulating surface with the middle phalanx is almost horizontal and flat.

The distal interphalangeal joint is bridged by a dorsal tendon attached to the Mm. *Extensores breves* (Burton, 1998) and by the ventral *Tendo Superficialis* attached to the flexor muscles (Manzano et al. 2007; Fig. 5B_II,III_). The extensor tendon consists of two strands connected via a thin collagenous sheath, which together span the distal epiphysis of the middle phalanx (referred to as the head of the middle phalanx), and connect dorsally to the distal phalanx, just distally of the base of the distal phalanx. The *Tendo Superficialis* runs ventral of the middle phalanx and splits below the intercalary element into two strands. These strands flatten out below the base of the distal phalanx, follow its curved ventral surface and connect distoventrally to its base. Whereas the *Tendo Superficialis* inserts nearly parallel to the surface of the base of the distal phalanx, the extensor tendon attaches at an angle of about 45°.

Two collagenous ligaments (referred to as collateral ligaments) strengthen the distal interphalangeal joint ventrolaterally (Fig. 5B_II,III_). Each collateral ligament attaches laterally to the head of the middle phalanx, traverses past the intercalary element towards the ventrolateral side of the base of the distal phalanx, and from there towards the dorsolateral side of the base of the distal phalanx. From proximal to distal, various side arms branch off from the collateral ligaments (Fig. 5B_II,III_): (i) several side arms run towards the ventral cutis of the middle digital segment, (ii) below the intercalary element, the two main arms connect dorsally of the flexor tendon with each other via a medial running side arm, which also connects to the intercalary element, and (iii) below the base of the distal phalanx some branches run towards the ventral collagen layer. Low contrasts between neighbouring collagen strands impede the exact identification of the trajectories and attachment points of the collateral ligaments and the ventral collagen layer.

### Internal structures of the digital tip

#### Ventral epidermis

**Figure 6:**
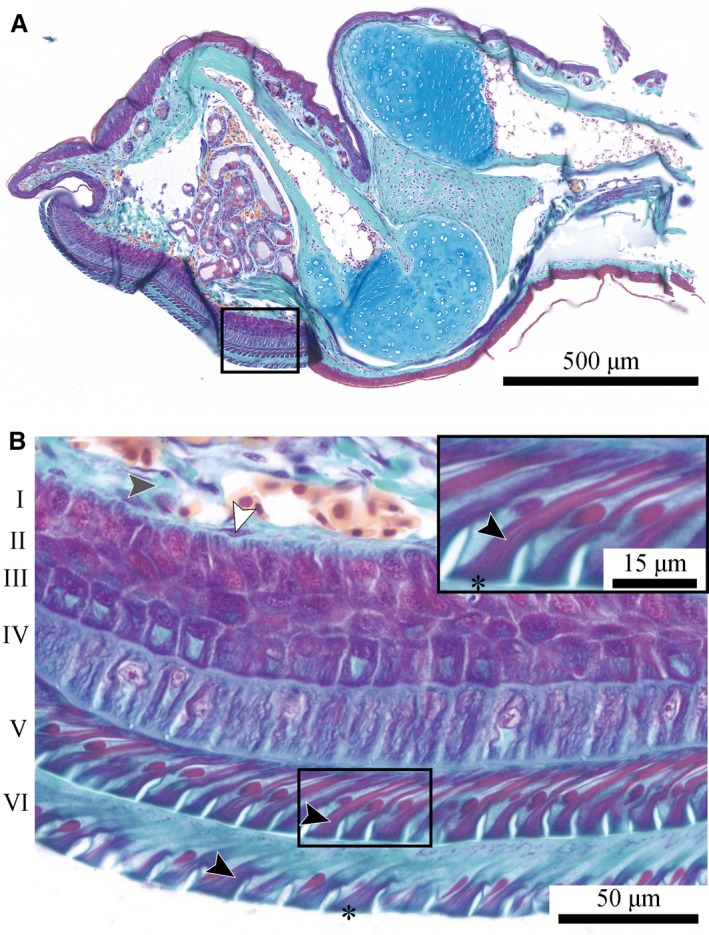
(A) Approximately mid‐sagittal section through a digital pad of *Hyla cinerea* (Frog 2, digit H_I_) stained with Crossmon's light green trichrome including Mayer's haematoxylin and Alcian blue. (B) Magnified view of the ventral epidermis [see box in (A)] containing tonofibrils (reddish; black arrowheads), reticular cells (white arrowhead) and reticular connective tissue (grey arrowhead). Layer numbering after Ernst (1973a).

The ventral epidermis is thicker than the dorsal one (Fig. 6A), stratified, and consists of up to six cell layers (Fig. 6B) numbered I–VI from basal to apical (Ernst, 1973a). Whereas the cells in layers I (i.e. the germinal layer) and II are shaped and arranged irregularly, the cells in layers III–VI are columnar. The cells of layers V and VI (i.e. the superficial layer) differ morphologically and arguably also functionally from the deeper cell layers: the cell bodies are skewed such that the apical cell surface is positioned distally to the basal one. Reddish staining indicates the presence of fibrous structures that connect broadly to the apical cell surface and merge into a thin bundle running towards the proximal‐basal cell surface. These structures are known to be tonofibrils (Ernst, 1973a; Nakano & Saino, 2016). The basal part of layer VI contains less tonofibrils than in layer V, presumably due to physiological changes impending ecdysis (Ernst, 1973a).

#### Collagenous structures

**Figure 7:**
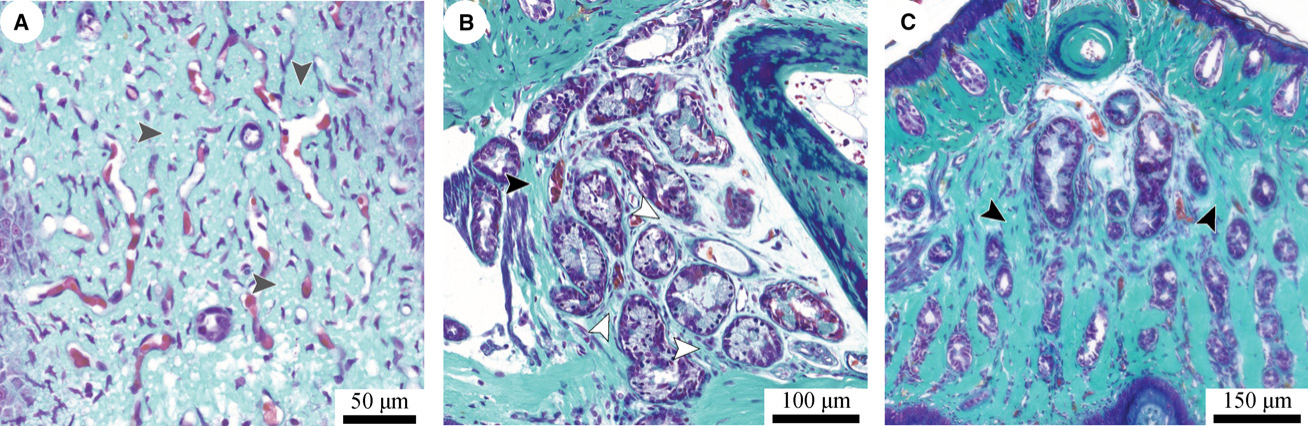
Collagen networks in a digital pad of *Hyla cinerea*. (A) Loose network of collagen fibres (light turquoise; grey arrowheads) in the ventral stratum spongiosum; horizontal section (Frog 3, digit F_I_). (B) Loose collagen network in the gland space (white arrowheads) and collagenous septum (black arrowheads); sagittal section (Frog 3, digit F_II_). (C) Septum (black arrowheads) separating the gland and lymph space; transverse section (Frog 3, digit F_III_). Transverse and sagittal sections are oriented upright; in sagittal and horizontal sections, the distal digit side is on the left and at the top, respectively.

The digital tip of *Hyla cinerea* includes several networks of collagen fibres with approximately isotropic fibre arrangements. The reticular collagen of the basement membrane (Fig. 6B) connects to the dermal stratum spongiosum (Fig. 3). Fine collagen fibres without a clear preferential orientation traverse the interglandular space. At the borders of the gland space, these fibres connect with the surrounding dermal connective tissue (Fig. 7B).

The pad also contains collagenous structures with distinctly anisotropic fibre arrangements. The thin septum, which compartmentalises the subdermal volume into the gland and lymph space (Fig. 7C), is fan‐shaped and consists of collagen fibres extending radially from the distal tip of the distal phalanx towards the ventral cutis. Collagen fibres connecting the dorsal and ventral cutis form the lateral fractions of the septum. The plane in which the septum fibres run is rotated by about 15–25° about the transverse pad‐axis, such that the dorsal tip of the septum is located distally from its ventral attachment.

**Figure 8:**
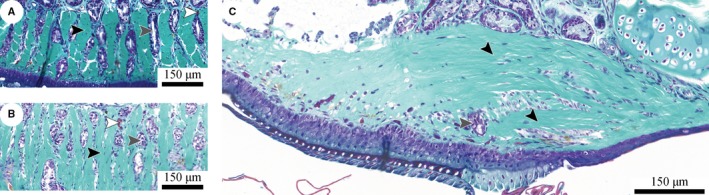
Ventral collagen layer and ridges (light turquoise; black arrowheads) in a digital pad of *Hyla cinerea* in (A) transverse (Frog 3, digit F_III_), (B) horizontal (Frog 3, digit F_I_) and (C) sagittal (Frog 3, digit F_II_) section. The ridges consist of collagen fibres curved around the lateral pad‐axis. The troughs between the ridges are filled with mucus ducts (grey arrowheads) and blood vessels (white arrowheads). Section orientations as in Fig. 7.

The ventral collagen layer is the most prominent collagenous structure in the digital pads of *Hyla cinerea* (Fig. 8). This layer fills the space between the ventral epidermis, gland space and ventral surface of the base of the distal phalanx, converges below this base, and connects to the side arms of the collateral ligaments, as described above. Whereas the collagen layer is only about 10–20 μm thick below the base of the distal phalanx, it reaches a thickness of up to 290 μm below the gland space. Distally of the septum, the layer thickness decreases towards the distal pad end (down to about 60–120 μm). At the location of minimum thickness below the base of the distal phalanx, the ventral collagen layer has a transversal cross‐sectional area in the order of 20 000–65 000 μm^2^ (measured for digits F_III_, H_II_ and H_V_ of Frog 3).

The ventral collagen layer consists of regular collagen fibres oriented along the distal‐proximal digit‐axis. As seen in sagittal sections (Fig. 8C), the fibre trajectories are curved around the transverse pad‐axis. The fibres are arranged in vertically separated bundles (Figs 2D and 8A) and connect approximately perpendicular with the ventral basement membrane. The distal extension of the collagen fibres increases from ventral to dorsal.

Below the gland space, the ventral collagen layer is laterally separated into about 15–20 longitudinal ridges divided by troughs (Figs 2B,C and 8A,B). These ridges are not present in the proximal, expanding part of the collagen layer, and they gradually flatten and vanish towards the distal end of the digital pad. The troughs contain mucus ducts and blood vessels running from the gland space towards the ventral pad surface.

#### Muscular structures

**Figure 9:**
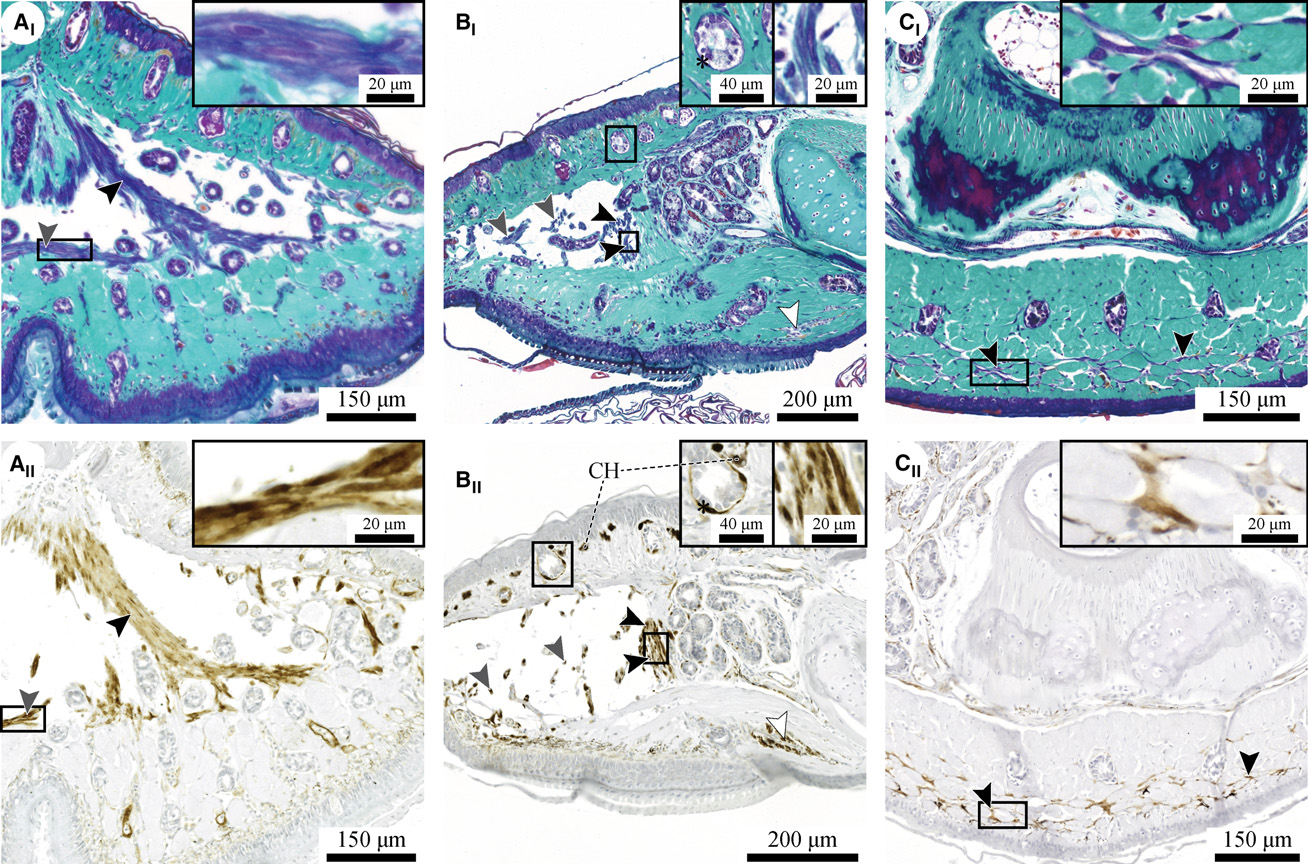
(I) Histochemically (purple‐dark blue) and (II) immunohistochemically (brown) stained smooth muscle fibres in a digital pad of *Hyla cinerea*. (A) Two thick muscle fibre bundles [black arrowheads in (A) and (B)] run from the distal tip of the distal phalanx towards the ventral epidermis, and distal‐cross‐lateral muscle fibres traverse the lymph space above the ventral dermis [grey arrowhead in (A)]; transverse section (A_I_: frog 3, digit F_III_; A_II_: frog 1, digit F_III_). (B) Thin muscle fibre bundles [grey arrowheads in (B)] traverse the lymph space ventrodorsally; sagittal section (B_I_: frog 3, digit F_II_; B_II_: frog 1, digit H_I_). Smooth muscle fibres also surround the mucus glands (stars). (C) A fine network of proximal‐cross‐lateral muscle fibres runs laterally through the ventral collagen layer below the widening base of the distal phalanx [black arrowhead in (C) and white arrowhead in (B)]; transverse section (C_I_: frog 3, digit F_III_; C_II_: frog 1, digit F_III_). Section orientations as in Fig. 7. CH, chromatophore.

The combination of histology, immunohistochemistry and μ‐CT reveals the presence of muscular structures in the digital pads of *Hyla cinerea* (Figs 5A and 9). Generally, these structures appear as (bundles of) long cellular fibres with diameters ≤ 5 μm containing elongated nuclei. Based on the positive immunohistochemical staining and the absence of striations, we identify the fibres as smooth muscle fibres.

Most muscle fibres are located in the distal lymph space. Two distinct bundles of muscle fibres (referred to as thick muscle fibre bundles) extend from the distal tip of the distal phalanx ventrolaterally towards the ventral cutis (Fig. 9A,B). More distally, numerous finer muscle fibre bundles (referred to as thin muscle fibre bundles) traverse the lymph space in between dorsal and ventral dermis (Figs 5A and 9B). These bundles are occasionally interconnected within the lymph space and are, in general, rotated about the transverse pad‐axis such that their dorsal attachment is located more proximally than their ventral attachment. The total horizontal cross‐sectional area of all (thin and thick) muscle fibre bundles traversing the lymph space dorsoventrally ranges approximately from 7000 to 14 000 μm^2^ (measured for digits F_I_ and H_V_ of Frog 3 and digit F_I_ of Frog 1). Additionally, several bundles of smooth muscle fibres run cross‐laterally through the lymph space close to the ventral dermis (referred to as distal‐cross‐lateral muscle fibre bundles; Fig. 9A). These bundles appear mostly in the proximal half of the lymph space and connect to the dorsoventrally oriented fibres.

Another fine network of smooth muscle fibres is present in the proximal, widening part of the ventral collagen layer below the base of the distal phalanx. These fibres (referred to as proximal‐cross‐lateral muscle fibre bundles) run cross‐laterally through the apical third to half of the collagen layer between the longitudinal bundles of collagen fibres. The position of this muscle fibre network along the longitudinal pad‐axis coincides with the proximal beginning of the pillar pattern on the ventral epidermis (Fig. 8C).

### Topologically optimised pad model under shear load

**Figure 10:**
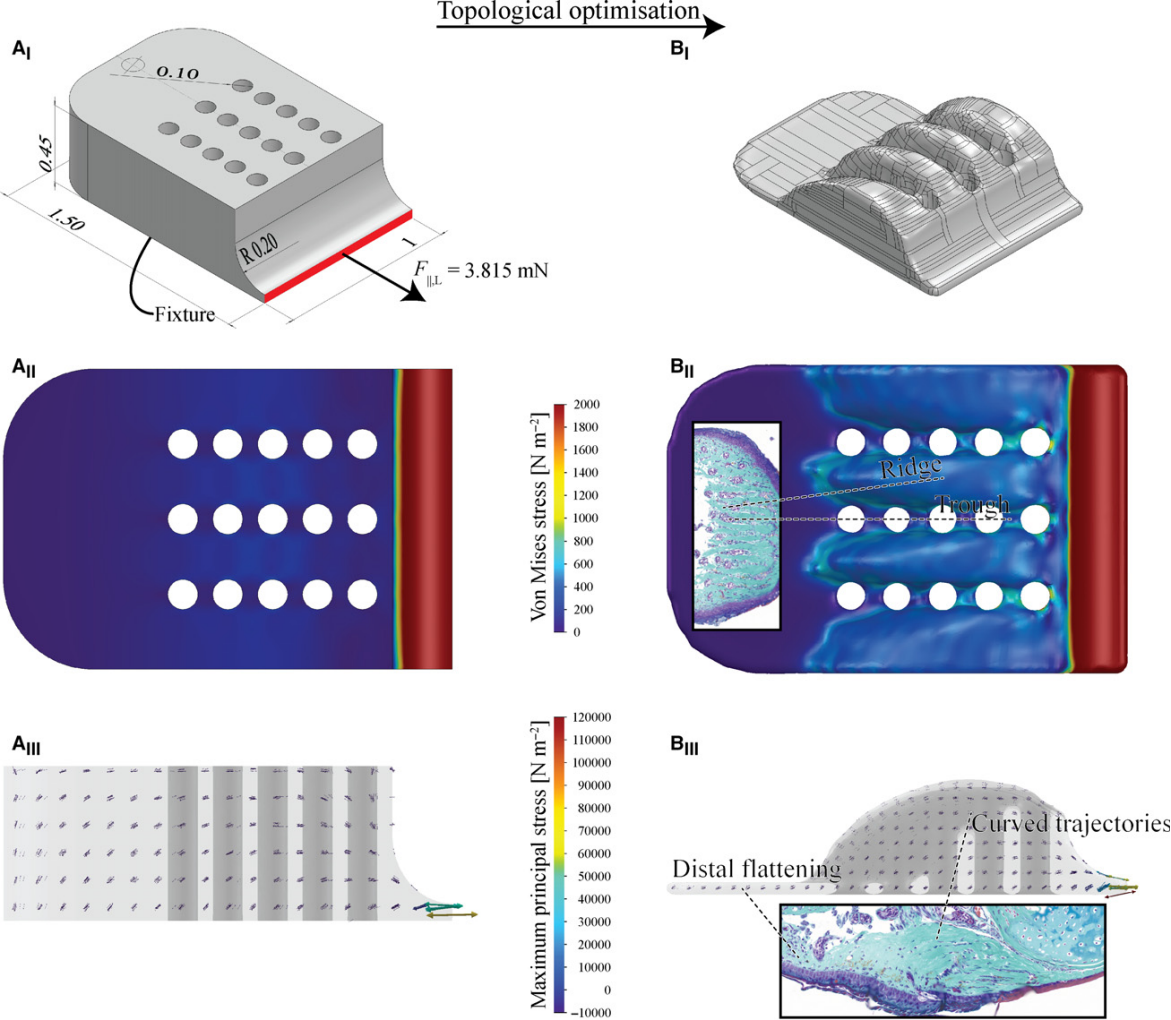
Topological optimisation of a shear‐loaded pad model. (A) Non‐optimised, ventrally fixed model (Young's modulus *E* = 20 MPa, Poisson's ratio *ν* = 0.33) with the approximate shape and size of the ventral collagen layer in a digital pad under a shear load F_II,L_ acting on the proximal surface (red). (B) Topological optimisation leads to the formation of longitudinal ridges, to distal flattening, and to curved stress trajectories, similar to the ridges and the distribution as well as orientation of the collagen fibres, respectively, in the ventral collagen layer in the digital pads of *Hyla cinerea* (see insets). (I) Geometrical models in dorsoproximal view. The non‐optimised model is dimensioned in mm. (II) Von Mises stresses (dorsal view) indicating regions of low mechanical loading. (III) Vector plot of the maximum principal stresses (lateral view) showing the trajectories of force transmission.

The FEA of a non‐optimised, shear‐loaded model of the ventral collagen layer in the digital pad of *Hyla cinerea* shows higher von Mises stresses between the longitudinal rows of holes piercing the model than in between the holes within a row (Fig. 10A_II_). Overall, von Mises stresses decrease from proximal to distal. The maximum principal stress trajectories are curved around the transverse model axis and run from the most proximal model surface towards the ventral surface (Fig. 10A_III_). The distal extension of the stress trajectories increases from ventral to dorsal.

Topological optimisation leads to distal flattening of the model and to the ‘carving out’ of longitudinal, curved ridges between the rows of holes. The ridges are separated by troughs running in line with the rows of holes (Fig. 10B_I_). Von Mises stresses are lower in the material separating the holes within a row than in between the rows (Fig. 10B_II_). The maximum principal stress trajectories follow the curved shape of the ridges (Fig. 10B_III_).

## Discussion

**Figure 11:**
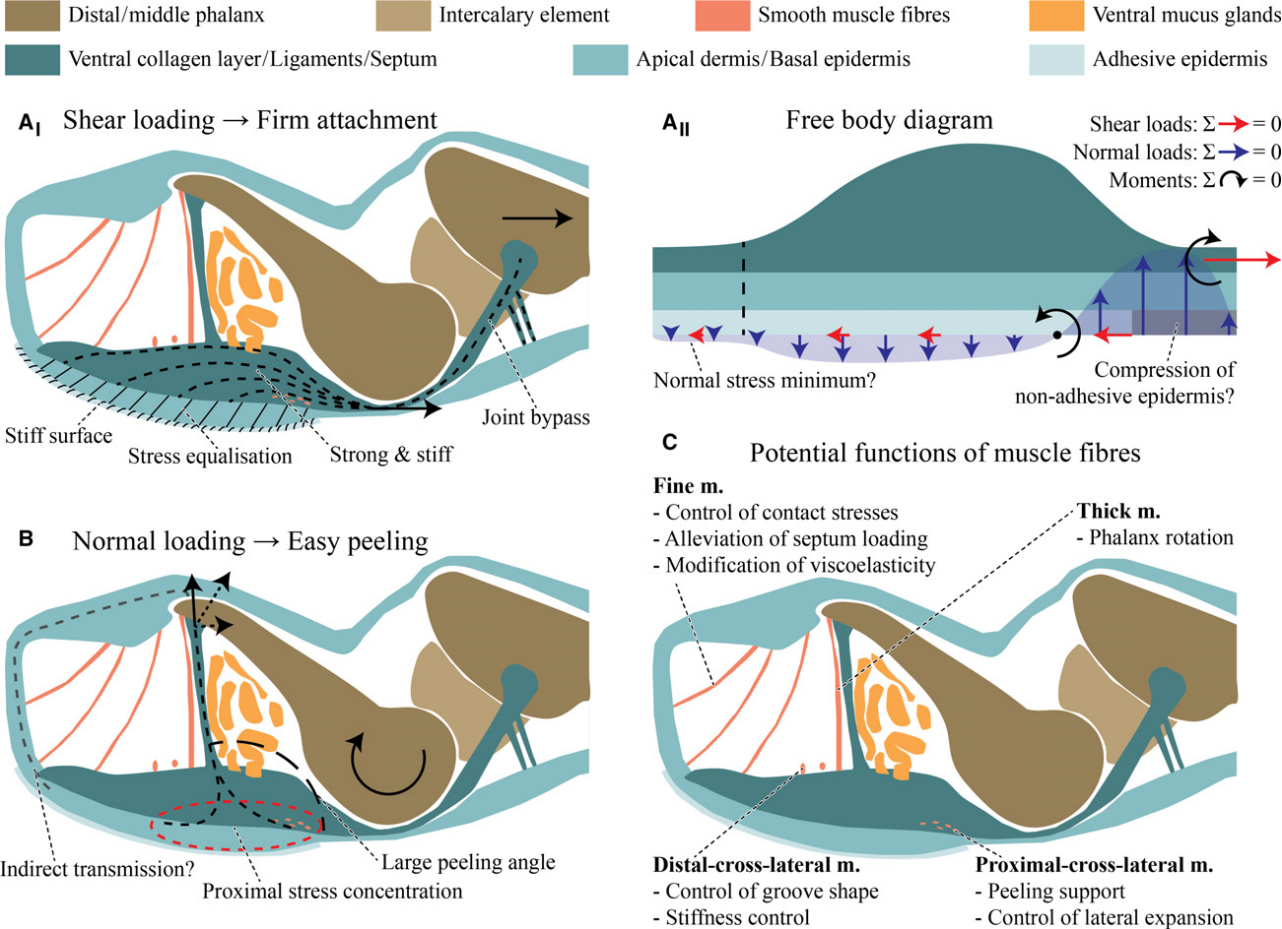
Schematic representation of a digital pad of *Hyla cinerea* in the midsagittal plane. (A_I_) Proposed mechanism of shear load transmission during proximal pulling on the middle phalanx. (A_II_) Equilibrium of the external forces and moments (free body diagram) acting on the shear‐loaded ventral cutis and collagen layer with a hypothetical distribution of shear and normal loads acting on the ventral pad surface during steady attachment. In reality, shear loads will be orders of magnitude higher than normal loads. (B) Hypothesised mechanism of normal load transmission and the induction of peeling during extension of the distal phalanx. (C) Proposed functions of the smooth muscle fibres (m.).

The dermal and subcutaneous structures in the digital pads of tree frogs have received little attention in previous research. To our knowledge, the latest reported efforts to analyse the internal morphology of the pads were made 90 years ago (Noble & Jaeckle, 1928), while later studies focussed on bony and cartilaginous structures (Paukstis & Brown, 1991; Manzano et al. 2007; Kamermans & Vences, 2009) and the superficial epidermis (Ernst, 1973a; Green, 1979; Green & Simon, 1986; Hertwig & Sinsch, 1995; Mizuhira, 2004; Scholz et al. 2009; Barnes et al. 2013; Nokhbatolfoghahai, 2013; Chakraborti et al. 2014a,b; Drotlef et al. 2015; Nakano & Saino, 2016). Overall, we find a number of tissues that were until now undescribed or have been characterised only partially. As we discuss below, some of these tissues clearly are involved in attachment, particularly offering pathways for the transmission of adhesive and frictional attachment forces. Although adhesion and friction are interdependent and some of the structures (e.g. the ventral epidermis) obviously are loaded during normal and shear loading, this may not be the case for all structures (e.g. the collateral ligaments). Moreover, some structures seem primarily involved in either normal (e.g. the septum) or shear loading (e.g. the ventral collagen layer). Therefore, we separately discuss the transmission of shear loads from the ventral epidermis via the ventral collagen layer to the collateral ligaments (Fig. 11A_I_), the transmission of normal loads through the septum (Fig. 11B), and potential functions of the smooth muscle fibres in the digital pads of tree frogs (Fig. 11C).

### Transmission of shear loads

During contact formation, mechanical loads are initially taken up by the ventral epidermis. Overall, the epidermal morphology found in this study agrees with the extensive description for the same species by Ernst (1973a). The presence of skewed cells in the second most apical layer of epidermal cells in *Hyla cinerea* (this study), *Litoria caerulea* (Nakano & Saino, 2016) and *Staurois parvus* (Drotlef et al. 2015) contradicts cell skewing to be a mere age effect, as suggested previously (Schuberg, 1891; Ernst, 1973a). We hypothesise that the longitudinally skewed tonofibrils increase the stiffness of the apical epidermis during the transmission of shear loads deeper into the digital pad, possibly increasing friction by the distribution of mechanical stresses over a larger volume of pad material (Xue et al. 2017) and maintaining the structural integrity of the epidermal surface. The dense ‘sponge‐like’ network of tonofibrils within the apical regions of the superficial cells may locally increase the mechanical resilience of the epidermis surface (Drotlef et al. 2015; Nakano & Saino, 2016). Moreover, the skewed tonofibrils may help to increase the pad‐substrate contact area and adhesion during proximal pulling of the pad (Drotlef et al. 2015), thus explaining the anisotropic friction measured in tree frogs (Hanna & Barnes, 1991; Chen et al. 2015). Anisotropic friction has also been reported for smooth insect pads containing cuticle fibrils (Bullock et al. 2008; Dirks et al. 2012), as well as the skewed fibrous attachment systems of geckos (Autumn et al. 2006) and technical adhesives (Murphy et al. 2007; Xue et al. 2014). Further work is needed to illuminate the function of the skewed tonofibrils in tree frog attachment.

The basal epidermal layers are in earlier stages of ecdysis than the apical ones and do not show the longitudinally skewed tonofibrils, as observed previously (Ernst, 1973a). We hypothesise that the basal epidermis forms a layer with (nearly) isotropic material properties, together with the approximately randomly oriented collagen fibres in the basement membrane and in the stratum spongiosum, which agree with general descriptions of these tissues in amphibians (Toledo & Jared, 1993; Haslam et al. 2014). A direct fibrous connection between the epidermal surface and the skeleton concurs with the risk of local overloading of the fibres or of the contact interface, causing local material failure or peeling, respectively. We hypothesise that the (nearly) isotropic layer serves in the horizontal distribution and equalisation of mechanical stresses taken up from the superficial epidermis, hence reducing the risk of local peeling or material damage (Fig. 11A_I_). Furthermore, deformations during loading arguably are higher in the softer basal epidermal layers than in the stiffer apical ones. Accordingly, the basal epidermis may contribute to viscoelastic shock absorption, as suggested for the strongly vascularised ventral stratum spongiosum (Barnes et al. 2011). Further work, for example immunohistochemistry and transmission electron microscopy, is required to analyse the molecular and structural basis of anchoring of the ventral collagen layer in the basement membrane.

The ventral collagen layer arguably takes up mechanical loads from the more ventral tissues. The high level of structural ordering and the strong connectivity with more ventral structures suggest that the collagen layer is the dermal stratum compactum (Toledo & Jared, 1993; Haslam et al. 2014). The laterally separated ridges in the ventral collagen layer in *Hyla cinerea* have been also reported for various other species (Noble & Jaeckle, 1928). Both the toe pad and the numerical model show a flat and relatively unperforated region distally of the (artificial) mucus ducts, laterally separated ridges in the duct region, and curved trajectories of the collagen fibres and the maximum principal stresses, respectively (Fig. 10B). These similarities between the morphology of the ventral collagen layer and the morphology predicted from the numerical pad model support our hypothesis that the collagen layer is adapted towards a high stiffness during proximal shear loading of the ventral pad surface. Material in the dorsodistal pad region contributes only little to the stiffness and hence is not required. Mucus glands, which are present in normal amphibian skin (Haslam et al. 2014), would weaken the collagen layer mechanically. We argue that the ‘outsourcing’ of the glands is a consequence of the need for a high stiffness/strength of the collagen layer. In turn, the ridges result from the need for ducts connecting the glands with the ventral pad surface. It is unavoidable that also the ducts locally weaken the collagen layer. The serial duct arrangement arguably agrees with the lowest possible loss of material in the transverse plane and accordingly with the highest possible stiffness for a given transverse cross‐sectional area. The material between the ducts within a row experiences relatively low mechanical stresses (Fig. 10A_II_,B_II_), contributes only little to the stiffness, and therefore is recessed in the numerical model. The structural ‘pre‐alignment’ of the unloaded collagen layer and epidermis suggests the importance of rapid mechanical functioning of these structures at the event of (shear‐)loading. Comparing the morphology of the whole digital pad for different load cases such as shear and (compressive and tensile) normal loading, as done for the superficial epidermis (Nakano & Saino, 2016), may help to quantify the amount of deformation of the various internal pad structures and to illuminate the attachment‐related functions of these structures.

The presence of ridges in the collagen layer is also practically relevant for future studies: the ridges presumably give the pad anisotropic material properties, with a lower stiffness in the transverse direction than along the longitudinal pad‐axis. Accordingly, the pad deformation during (de‐)hydration, for example by vacuumisation in electron microscopy, may be anisotropic, which should be considered in the quantification of geometrical parameters of the superficial epidermal cells. In microindentation‐studies, variations of pad parameters such as stiffness and work of adhesion (Barnes et al. 2011) may be partially related to the location of the indentation with respect to the ridges.

Proximally, the ventral collagen layer connects via the collateral ligaments with the middle phalanx, as described by Noble & Jaeckle (1928). We propose that these ligaments represent a bypass in force transmission around the distal joint complex: a shear load acting on the ventral collagen layer is transmitted via the ligaments directly to the middle phalanx and further. Hence, force transmission is achieved quickly and without the need of stabilising the distal phalanx by muscle activity. The ligament side arms connecting to the ventral cutis of the middle digital segment, which were observed also in *Osteopilus septentrionalis* (Noble & Jaeckle, 1928), may provide additional proximal ‘anchor points’ for the collateral ligaments, hence supporting the ligaments in load transmission.

Overall, the structures connecting the ventral pad surface with the middle phalanx are presumably adapted towards a high stiffness and strength as well as the equalisation of local stresses during shear loading (Fig. 11A_I_). For example, we estimate that the ventral collagen layer can withstand a tensile load of 2.0–6.5 N before material failure, based on the measured transverse cross‐sectional area and assuming a tensile strength of 100 MPa (Biewener, 2008). This is well above the maximum load of 1.27 N measured for single digital pads of *Trachycephalus resinifictrix* (Bijma et al. 2016). Considering peak shear stresses of up to 70 kPa (*Rhacophorus dennysi*; Endlein et al. 2017) and 140 kPa (*Litoria caerulea*; Crawford et al. 2016) withstood by the epidermal surface, and the high tensile strength of the collagen layer, we argue that the digital pads are adapted primarily towards the generation and transmission of frictional rather than adhesive forces. This agrees with the functional demands on the pad arising from the locomotion and habitat of tree frogs. During jumping and landing, terrestrial frogs regularly experience ground reaction forces in the range of 1–5 N (Nauwelaerts & Aerts, 2006). Frictional pad loading might be even higher and a high pad strength even more important when a tree frog falls in its arboreal habitat and has to hold on to a leaf or twig (Bijma et al. 2016) to avoid death. Next to frictional forces, it is unavoidable that adhesive forces also act on the ventral pad surface during shear loading (Fig. 11A_II_): a proximal shear load acts not within the contact interface, but more dorsally on the load‐transmitting structures, hence creating a moment acting on the ventral pad surface. The generation of (compressive and tensile) normal attachment forces at the pad surface is unavoidable to counteract this moment during steady attachment (Fig. 11A_II_). The spatial distribution of the normal mechanical stresses is still unclear, but they are arguably lower in magnitude than the occurring shear stresses.

Importantly, tree frogs have been observed to pull their digital pads proximally over the substrate after contact formation (Schuberg, 1891; Hanna & Barnes, 1991); this movement may also occur when pulling proximally on the flexor tendon (Schuberg, 1891). Endlein et al. (2017) measured increased adhesion and friction as a consequence of such pulling movements. In geckos and gecko‐inspired adhesives (Bartlett et al. 2012), a similar correlation between attachment force and shear loading has been assigned to a scaling of the attachment force with the effective stiffness of the according adhesive systems. Although the physics of the scaling of attachment force with material stiffness are still under debate (Mojdehi et al. 2017), the presence of a stiff load‐transmitting structure connecting the adhesive surface with the skeleton both in the ‘dry’ digital pads of geckos (Russell, 1986) and the ‘wet’ pads of tree frogs suggests functional relevance of these internal structures in attachment. In general, an adhesive should be soft to facilitate the conformation to the substrate and the enlargement of the contact area (Bartlett et al. 2012). During adhesive loading, however, a stiff material arguably promotes the rapid formation of a mechanical link between contact surface and substrate. Furthermore, a stiff material may facilitate the spatial distribution of contact stresses and hence could impede detachment. The digital pads of tree frogs (Fig. 11A_I_) that are soft in normal loading (Barnes et al. 2011, 2013; Kappl et al. 2016) and stiff in shear loading (as suggested in this study) might make use of these effects. As discussed above, the basal epidermal cell layers form a nearly isotropic (and potentially soft) link in the transmission of shear loads, which may help to reduce peak loads. The epidermis is, however, quite thin. Also, the basal epidermal cells are interdigitated and connected via desmosomes with the basal dermal structures as well as with the more distal epidermal cells (Ernst, 1973a). Hence, the basal epidermis presumably does not have a strong adverse effect on the shear stiffening discussed here. Simultaneous measurements of the elastic modulus, compliance and attachment force as well as a quantification of the spatial contact stress distribution at the pad‐substrate interface are needed to analyse the shear stiffening of the digital pads of tree frogs.

### Transmission of normal loads: the septum as an adaptation towards peeling?

As in shear loading, the normal adhesive loads acting on the ventral pad surface must be transmitted through the cutaneous and subcutaneous structures to the internal skeleton. As discussed above, the ventral cutis seems to be adapted primarily towards the transmission of shear rather than normal loads: the naturally skewed trajectories of the tonofibrils in the superficial epidermal cells negate potential adhesion‐enhancing effects found in tree‐frog‐inspired adhesives including normally oriented fibres (Xue et al. 2017).

Load transmission from the ventral cutis to the distal phalanx could be achieved by the collagenous septum separating the distal lymph space and the proximal gland space, and by the (thick and thin) smooth muscle fibre bundles traversing the lymph space dorsoventrally (Fig. 11B). To our knowledge, the septum has not been described previously, although Schuberg (1891) mentioned the thick muscle fibre bundles that reinforce the septum distally. Clearly, the septum functions as a separating wall between gland and lymph space. However, it is unclear if the septum mainly serves for clustering the glands or for creating a ‘free’ lymph space. The septum has a horizontal cross‐sectional area of about 6500–7500 μm^2^ (measured for digits F_III_ of Frog 1 and H_V_ of Frog 3), which corresponds with a maximum tensile load before material failure of 0.65–0.75 N, assuming the same tensile strength as for the ventral collagen layer. In total, the dorsoventral muscle fibre bundles can only bear a drastically lower load of 2.8–5.6 mN (assuming a tensile strength of 0.2–0.4 MPa of skeletal muscle; Biewener, 2008), which suggests that these muscle fibre bundles (if involved at all) only have a supportive role in normal force transmission. In total, the muscle‐collagen‐complex should be well able to withstand the fraction of the approximate body weight acting on a single digit (i.e. 3.815 mN). However, (i) the tensile strength and the cross‐sectional area mentioned above may have been overestimated for the relatively loose collagenous septum, and (ii) it is unclear how loads are distributed between the stronger septum and the weaker muscle fibre bundles.

The muscle‐septum‐complex connects with the ventral cutis along a cross‐lateral line that results from the intersection of the horizontal dermal plane and the septum plane. This locally confined conjunction of septum and cutis implies a local concentration of mechanical stresses during dorsodistal pulling on the septum as a result of extension or translation of the distal phalanx, which increases the probability of peeling at the proximal pad surface (Fig. 11B). In fact, a simplified FEA model shows (i) the highest normal contact stress at the proximal edge of the ventral contact surface of a pad model when loading the septum dorsodistally and (ii) a higher averaged normal contact stress when loading the pad model via the septum compared with loading via the proximal end surface of the pad model (see Appendix S8). Moreover, dorsodistal pulling on the septum coincides with a large peeling angle (> 90°) relative to the substrate, reducing the pulling force needed for peeling according to peeling theory (Kendall, 1975). The induction of peeling by pulling proximally (via the collateral ligaments) on the ventral collagen layer, as suggested by Hanna & Barnes (1991), requires either a higher pulling force than for pulling on the septum, or an increase of the peeling angle by forward‐rotation of the whole digit. Overall, these observations support the hypothesis that the septum represents an adaptation towards (rapid and efficient) detachment rather than attachment (Fig. 11B). Peeling‐induction via the septum may be especially important for the detachment of tree frogs in their typical resting position, when the limbs are positioned closely below the body (own observations, but also Siedlecki, 1909; Endlein et al. 2012) and large‐scale limb movements may be hindered in escape manoeuvres, when fast detachment is decisive, or during locomotion, when detachment should be as energy‐efficient as possible. The hypothesised role of the septum in detachment could be tested, for example, by comparing the pull‐off forces of an unmodified digital pad and a pad with disjoint septum; if peeling occurs via septum peeling, the original pad should detach more easily than the manipulated one.

Alternatively, normal force transmission might be achieved indirectly via the dorsal and lateral cutis. In *Hyla arborea* and *Rhacophorus reinwardtii*, the distal phalanx is known to bulge out the dorsal cutis (Schuberg, 1891; Siedlecki, 1910), which is also seen in the segmented digital tip of *Hyla cinerea* (Fig. 5A). In indirect force transmission, contact stresses would be automatically concentrated at the edge of the contact surface, making peeling more likely. If present at all, indirect force transmission is less favourable than a direct one.

### Attachment‐related functions of the smooth musculature

We find several muscular structures in the digital pads of *Hyla cinerea* that are not mentioned in the current literature (Ernst, 1973b). The primary locomotory musculature is located more proximally (Manzano et al. 2008) and the muscle fibres in the digital tip most likely facilitate fine modulations of the digital tip (Fig. 11C).

The two thick bundles of smooth muscle fibres mentioned above, which have been observed also in *Hyla arborea* (Schuberg, 1891), connect the distal tip of the distal phalanx with the ventral cutis. Schuberg (1891) argues that the muscle fibre bundles support the flexion of the distal phalanx, and hence the exertion of pressure on the gland space and the secretion of mucus. The large moment arm created by the attachment of these muscle fibre bundles at the distal end of the distal phalanx with respect to the distal joint supports a function in phalanx flexion.

The thin bundles of smooth muscle fibres traversing the lymph space dorsoventrally have been described previously for *Hyla arborea* as connective tissue (v. Wittich, 1854; Leydig, 1868; Dewitz, 1883), although their muscular nature was later confirmed (Schuberg, 1891). The μ‐CT data show that these thin muscle fibre bundles do not connect to the distal tip of the distal phalanx, as stated previously, but to the dorsal dermis. Therefore, they presumably do not fulfil the same function as the thick muscle fibre bundles. Instead, the concerted activation and contraction of the thin fibres might enable the spatial control of contact stresses at the pad‐substrate interface, thus promoting or avoiding peeling. Moreover, contraction of the thin muscle fibre bundles might alleviate the mechanical stresses in the two thick muscle fibre bundles and the septum, hence avoiding peeling via septum‐loading as discussed above. Additionally, contraction of the fibres in the lymph space might allow for the active modification of the stiffness and hence the contact area (Afferante et al. 2016), or of the viscoelasticity of the lymph space, which could act as macroscopic shock absorber with muscle‐modulated dampening properties.

Distal‐cross‐lateral muscle fibres have also been found in other hylids by Schuberg (1891) and Gadow (1909), who suggested that these fibres control the shape of the longitudinal macroscopic grooves (Ohler, 1995; Lee et al. 2001; Nokhbatolfoghahai, 2013) observed in the ventral pad surface.

The (to our knowledge previously undescribed) proximal‐cross‐lateral muscle fibres described above might aid in controlling the lateral expansion of the ventral collagen layer during normal loading and hence the secretion of mucus. Alternatively, contraction of these fibres might increase contact stresses at the proximal edge of the contact surface. As discussed above, the pad starts peeling off proximally during regular walking (Hanna & Barnes, 1991), and local contact stress enhancement might ease this peeling motion.

Further research is needed to conclude on the function(s) of the smooth muscle fibres. Observing geometrical changes in the digital pad during local *in vivo in situ* electrostimulation of muscular or neural structures may help to elucidate the mechanical function of the contraction of specific muscle fibre groups. As a first step towards such an experiment, we suggest studying the innervation of the muscular structures in the digital tip (e.g. Is the nervous system sympathetic or parasympathetic? Are the neural and muscular structures arranged in motor units?). Clarifying the function(s) of the muscle fibres is not only relevant from a biomechanical viewpoint; understanding the insertion of the muscle fibres at the distal phalanx may also generate new impulses in the phylogenetic classification of tree frogs based on the phalangeal morphology (Kamermans & Vences, 2009). The presence of muscular structures also has practical consequences: the (hemi‐)spherical shape assumed in current contact mechanics models applied to tree frog attachment (e.g. the Johnson‐Kendall‐Roberts model; Johnson et al. 1971) may be an oversimplification of the digital pads of tree frogs, as these animals are potentially able to actively modulate the pad geometry as well as the distribution of contact stresses at the contact interface. In addition to common artefacts such as swelling/shrinkage of the samples, measurements on anaesthetised or euthanised animals may come with morphological artefacts due to the relaxed state of the muscles, that do not occur during *in vivo* measurements.

## Conclusions

1. The 3‐D morphological analysis of the digital pads of the tree frog *Hyla cinerea* reveals a general bauplan, which comprises, among others, a ventral collagen layer, collateral ligaments, a septum compartmentalising the subcutaneous volume into a distal lymph space and a proximal gland space, and muscular structures.
2. The ventral collagen layer consists of longitudinally oriented collagen fibres and forms, together with collateral ligaments, a mechanical link between the ventral pad surface and the middle phalanx during shear loading. Similarities in the morphology of the ventral collagen layer and a numerically optimised model suggest that the collagen layer is primarily adapted towards the transmission of shear loads. We estimate that the collagen layer can withstand a shear load of up to 6.5 N.
3. The septum forms a mechanical link between the ventral cutis and the distal tip of the distal phalanx. Further work is required to test our hypothesis that dorsodistal pulling of the septum facilitates proximal peeling of the pad.
4. The digital pads of *Hyla cinerea* (and of other species) contain smooth muscular structures besides the myoepithelial cells around the mucus glands. Numerous thin muscle fibre bundles and two thick muscle fibre bundles traverse the lymph space dorsoventrally. Furthermore, several muscle fibre bundles run cross‐laterally through the lymph space as well as though the proximal‐apical part of the ventral collagen layer. Further work is needed to conclude on the attachment‐related function(s) of these muscular structures.
5. Overall, this study adds to the knowledge on the digital pads of tree frogs, hence offering novel perspectives on the ecology, phylogeny and evolution of anurans, as well as contributing to the functional understanding of tree frog attachment: we expect that the digital pads of tree frogs will provide inspiration for the design of biomimetic adhesives beyond geometrical modifications of the contact surface.

## Author contributions

Conception of the study: JKAL, HS, JLvL; data acquisition: JKAL, HS, AB, FvdB, SWSG; data analysis: JKAL, HS; data interpretation: JKAL, JLvL, HS, SWSG; drafting of the manuscript and figures, literature analysis: JKAL; critical revision of the manuscript and the figures: JKAL, JLvL, SWSG, HS; final approval of the article: all authors.

## Supporting information


**Appendix S1.** Symbols and abbreviations.
**Appendix S2.** Housing conditions.
**Appendix S3.** μ‐CT image analysis.
**Appendix S4.** Histochemical protocols.
**Appendix S5.** Immunohistochemical protocols.
**Appendix S6.** Interdigital, interlimbal, interindividual and intermethodological comparision of the internal pad morphology.
**Appendix S7.** Intermediate topologically optimised geometries.
**Appendix S8.** Modelling of the normal contact stresses during peeling.

**Video S1.** The digital tip and internal structures of *Hyla cinerea* (Frog 3, digit H_V_) in dorsolateral view rotated by 360° around the dorsal‐ventral pad axis. Colour coding of the structures as in Fig. 5.

**Video S2.** The digital tip and internal structures of *Hyla cinerea* (Frog 3, digit H_V_) in ventrolateral view rotated by 360° around the dorsal‐ventral pad axis. Colour coding of the structures as in Fig. 5.
