# Supplementary material for: Force‐transmitting structures in the digital pads of the tree frog Hyla cinerea: a functional interpretation
Source: J Anat. 2018 Aug 19;233(4):478–95. doi: 10.1111/joa.12860 (PMC6131963; doi:10.1111/joa.12860)
Supplement: Supplementary file 1 — Appendix S1. Symbols and abbreviations. Appendix S2. Housing conditions. Appendix S3. μ‐CT image analysis. Appendix S4. Histochemical protocols. Appendix S5. Immunohistochemical protocols. Appendix S6. Interdigital, interlimbal, interindividual and intermethodological comparision of the internal pad morphology. Appendix S7. Intermediate topologically optimised geometries. Appendix S8. Modelling of the normal contact stresses during peeling. [file JOA-233-478-s001.pdf]

Force-transmitting structures in the digital pads of the tree frog *Hyla cinerea*: a functional interpretation

Supplementary material

Julian K.A. Langowski[1], Henk Schipper[1], Anne Blij[1], Frank T. van den Berg[1], Sander W.S. Gussekloo[1], Johan L. van Leeuwen[1]

[1] Experimental Zoology Group, Wageningen University & Research, Wageningen, The Netherlands

Correspondence
Julian K.A. Langowski, Experimental Zoology Group, Wageningen University & Research,
De Elst 1, 6708 WD Wageningen, The Netherlands.
E: julian.langowski@wur.nl

SI.1 Symbols and abbreviations

SI.1.1 List of symbols

Table SI.1: List of Roman (top) and Greek (bottom) symbols in alphabetical order.

Symbol	SI Unit	Description
E	Pa = kg m^{-1} s^{-2}	Young's modulus
$F_{\parallel,\mathrm{L}}$	N = kg m s^{-2}	Shear load
x	m	Longitudinal spatial coordinate
y	m	Lateral spatial coordinate
z	m	Vertical spatial coordinate
ν	-	Poisson's ratio
σ	Pa = kg m^{-1} s^{-2}	Yield strength
$\overline{\sigma_\perp}$	Pa = kg m^{-1} s^{-2}	Mean normal contact stress

SI.1.2 List of abbreviations

Table SI.2: List of abbreviations of morphological (top) and other (bottom) terms in alphabetical order.

Abbreviation	Description
BV	Blood vessel
ca.	circa
CH	Chromatophore
CO	Collagen tissue
DE	Dermis
DP	Distal phalanx
DU	Mucus duct
ED	Epidermis

ET	Tendon of the extensor muscle
F	Forelimb
FT	Tendon of the flexor muscle
H	Hindlimb
IE	Intercalary element
LI	Ligament
LY	Lymph space
m. / mm.	Muscle / muscles
MG	Mucus gland
MP	Middle phalanx
SE	Septum
SM	Smooth muscle
PB	Base of the distal phalanx
PH	Head of the middle phalanx
FEA	Finite Element Analysis
µ-CT	Synchrotron micro-computer-tomography
PTA	Phosphotungstic acid
stl	SurfaceTessellationLanguage

SI.2 Housing conditions

The animals were housed in $0.6 \cdot 0.6 \cdot 1.2$ m^3 (width · length · height) large terraria, with six frogs per terrarium, at the CARUS research facility at WUR. The terraria were enriched with plants (*Ficus spec.*) and a scaffold of polypropylen-pipes. The temperature was kept at 24–26 °C with heating mats and the relative air humidity was kept at 45–85% with a semi-automatised sprinkler system using demineralised water (Bitter Watertreatment, Netherlands). The frogs were kept at a 12 h : 12 h dark-light-cycle and fed 2–3 times per week with 3–5 live crickets enriched with vitamin/mineral powder (Dendrocare, AmVirep, Netherlands) per individual; water was supplied *ad libitum*. The room air was filtered for pathogens using an air purifier (WINIX U300, Winix, USA). The frogs were monitored daily for feeding state and abnormal behaviour.

SI.3 µ-CT image analysis

1) Median filter
    a. Radius = 1
2) Grey-value thresholding
    a. Filter band: 32772–35779
3) Histogram equalisation
    a. Equalisation = 1
    b. Number of bins = 100
    c. Number of ignored bins = 10

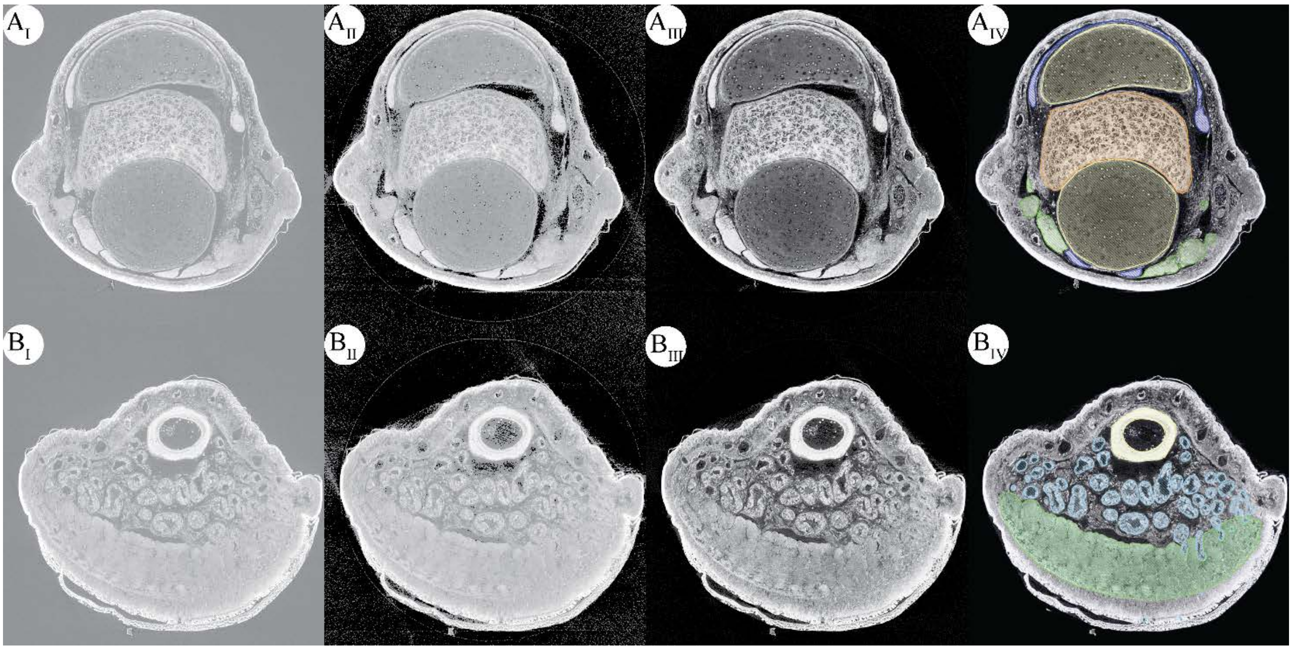

Fig. SI.1. The steps of image processing for transverse sections through a digital pad of *Hyla cinerea* (A) in the joint region and (B) through the digital gland space. (I) Original image, (II) background removal by grey-value-thresholding, (III) histogram equalisation, and (IV) segmentation (yellow: distal and middle phalanx, orange: intercalary element, light blue: ventral mucus glands, dark blue: tendons of the flexor and extensor muscles, green: ligaments and ventral collagen layer.

SI.4 Histochemical protocols

SI.4.1 Embedding protocol

1) Fixation and storage (see main text)
2) Rehydration
   a. Transfer of sample to 35% ethanol
   b. 2 min in distilled water
   c. Short submerging of sample in lukewarm 1% agarose gel
3) Agarose embedding
   a. Orientation of sample in lukewarm 1% agarose gel using a SZ-4 stereo microscope (Olympus, Japan)
   b. Hardening of agarose on a cold glass plate
   c. Cutting of agarose block according to desired cutting plane
4) Tissue-processing
   a. Dehydration

i. 70% ethanol	overnight	
ii. 80% ethanol	1	h
iii. 90% ethanol	45	min
iv. 96% ethanol	30	min
v. 100% ethanol	20	min
vi. 100% ethanol	20	min

   b. Cleaning

i. Xylene bath	$3 \cdot 1$	h

5) Paraffin embedding

a. Paraffin (KP Paraclean)	1	h
b. Vacuum oven	$3 \cdot 15$	min

SI.4.2 Staining solutions

Acetic acid (3%)

Solution A

Acetic acid, glacial	3	mL
Distilled water	97	mL

Alcian blue (pH 2.5)

Solution B

Acetic acid, glacial	3	mL
Distilled water	97	mL
Alcian Blue GX	1	g

Haematoxylin

Solution C

Haematoxylin	1	g
Distilled water	1000	mL
Sodium iodate	0.2	g
Potassium Alum	50	g
Chloral hydrate	50	g
Citric acid	1	g

Crossmon’s trichrome, light green

Solution D: Fuchsin/Orange G

Acid fuchsin	1.3	g

Orange G	1	g
Acetic acid, glacial	5	mL
Distilled water	500	mL
Thymol	0.33	g
**Solution E: Phosphomolybdic acid (5%)**		
Phosphomolybdic acid	25	g
Distilled water	500	mL
**Solution F: Light green (1%)**		
Light green SF	10	g
Acetic acid, glacial	10	mL
Distilled water	1000	mL

SI.4.3 Staining protocol

1) Deparaffinisation and rehydration to distilled water		
2) Solution A	3	min
3) Solution B	30	min
4) Rinsing in Solution A	20–30	s
5) Rinsing in distilled water	2 · 2	min
6) Solution C	7	min
7) Rinsing in running tap water	10	min
8) Solution D	1	dip
9) Rinsing in distilled water	2	min
10) Solution E	4	min
11) Rinsing in distilled water	2	min
12) Solution F	10	min
13) Rinsing in distilled water	2 · 1	min
14) Washing in 100% ethanol	4 · 2	min
15) Clearing with Xylene	3 · 2	min
16) Mounting with DPX		

SI.5 Immunohistochemical protocols

SI.5.1 Staining solutions

Peroxidase removal and blocker

Solution A: TBS-triton

Sodium chloride	8.8	g
Tris(hydroxymethyl)aminomethane (TRIS)	6.06	g
Triton 10%	2.5	mL
Hydrochloric acid (HCl)	until pH = 7.4 is reached	
Distilled water	fill up to 1000 mL	

Solution B: TBS-triton / H_2O_2

TBS-triton	222.75	mL
H_2O_2 30%	2.25	mL

Solution C: Blocker - goat serum 10% / BSA 1%

TBS-triton	3200	µL
Goat serum	400	µL
BSA-c 10%	400	µL

Solution D: Goat serum 2%

TBS-triton	8820	µL
Goat serum	180	µL

Antibodies

Solution E: 1st antibody – anti-α-smooth-muscle-actin (1:400)

Goat serum 2%	3990	µL
anti-α-smooth-muscle-actin	10	µL

Solution F: 2nd antibody – goat-anti-mouse/HRP (1:100)

Goat serum 2%	3960	µL
GaM/HRP	40	µL

DAB-stain

Solution G: TRIS-HCl

TRIS	6.06	g
HCl	until pH = 7.6 is reached	
Distilled water	fill up to 1000 mL	

Solution H: 3,3'-diaminobenzidine(DAB)-stain

TRIS-HCl	4500	µL
DAB stock (5 mg mL^{-1})	500	µL
H_2O_2 30%	5	µL

SI.5.2 Staining protocol

1) Deparaffinisation and rehydration to distilled water		
2) Solution B	20	min
3) Washing in solution A	2 · 5	min
4) Blocking with solution C in solution A	30	min
5) Incubation in solution E	overnight at 4 °C	
6) Washing in solution A	3 · 5	min
7) Incubation in solution F	45	min
8) Washing in solution A	2 · 5	min

9)	Washing in solution G	2 · 5	min
10)	Staining with solution H	8	min
11)	Rinsing in running tap water	10	min
12)	Mayer’s haematoxylin staining	1	min
13)	Rinsing in running tap water	10	min
14)	Dehydration (70% to 100% ethanol)		
15)	Clearing with Xylene	3 · 2	min
16)	Mounting with DPX		

SI.6 Interdigital, interlimbal, interindividual, and intermethodological comparisons of the internal pad morphology

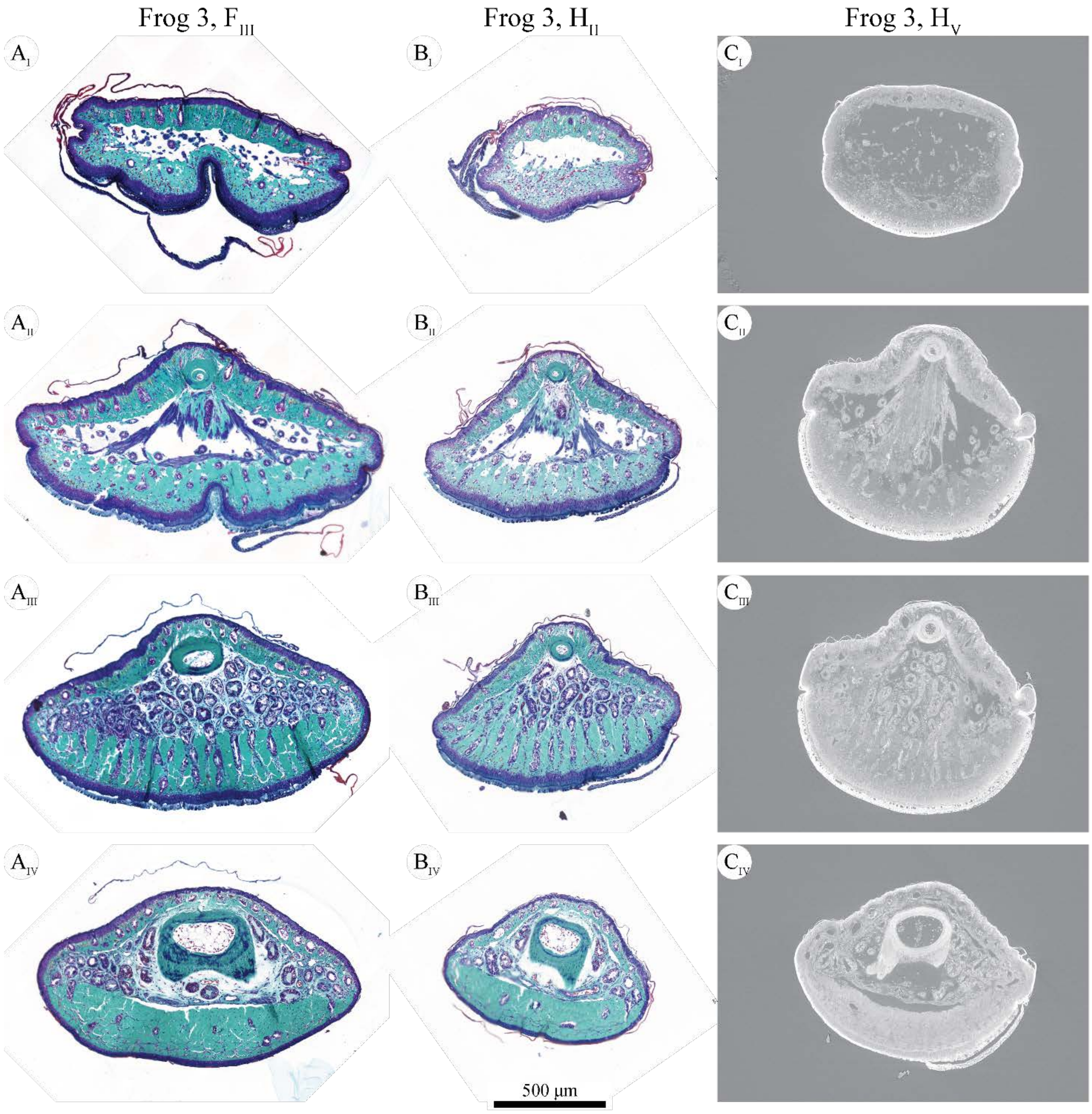

Fig. SI.2. Series of transverse sections through digital pads of the (A) fore- and (B,C) hindlimbs of *Hyla cinerea* obtained by (A,B) histology and by (C) µ-CT, from distal to proximal. The histological sections were stained with Crossmon's light green trichrome including haematoxylin and Alcian blue.

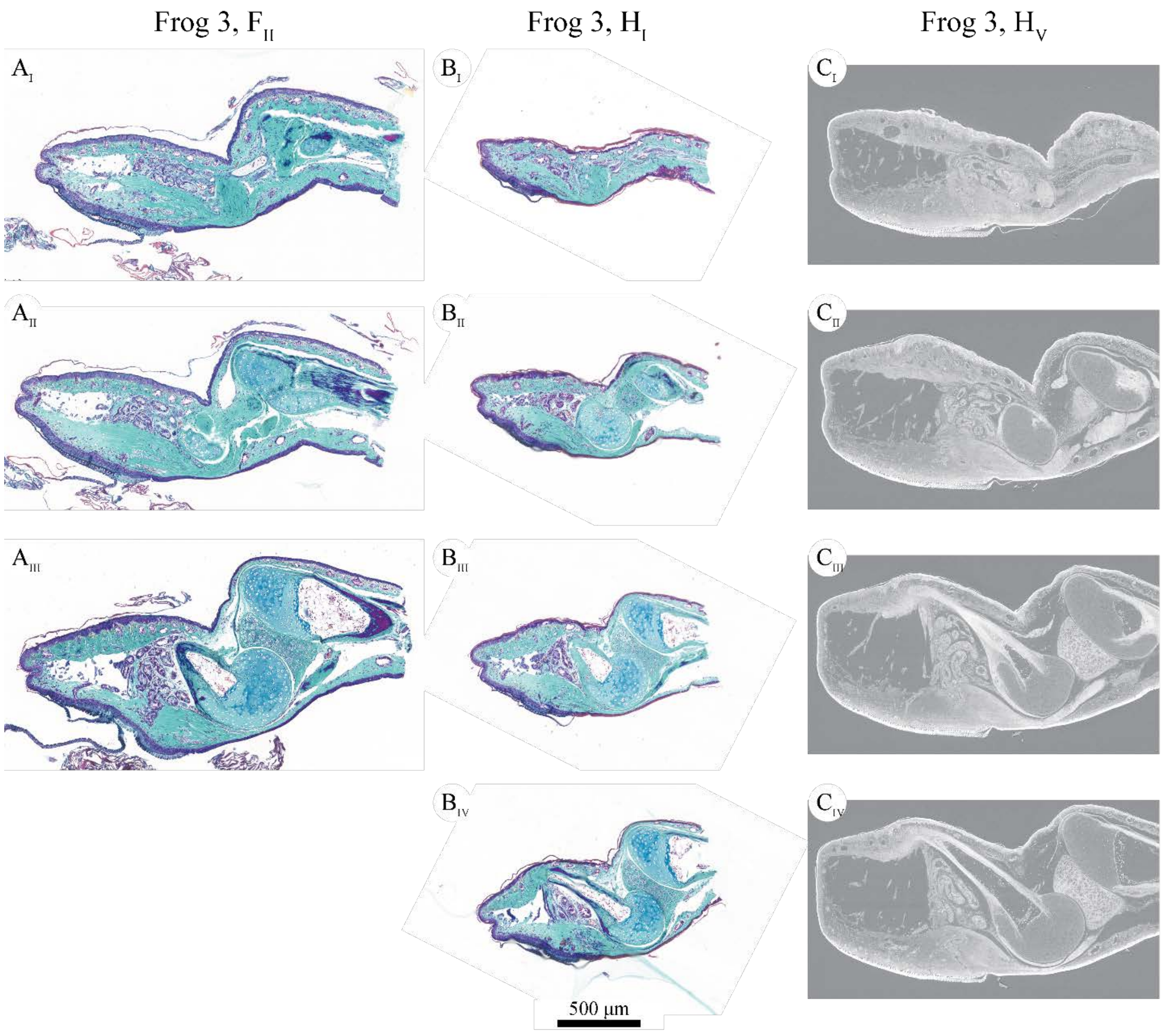

Fig. SI.3. Series of sagittal sections through digital pads of the (A) fore- and (B,C) hindlimbs of *Hyla cinerea* obtained by (A,B) histology and by (C) µ-CT, from lateral to mid-sagittal. The histological sections are stained with Crossmon's light green trichrome including haematoxylin and Alcian blue.

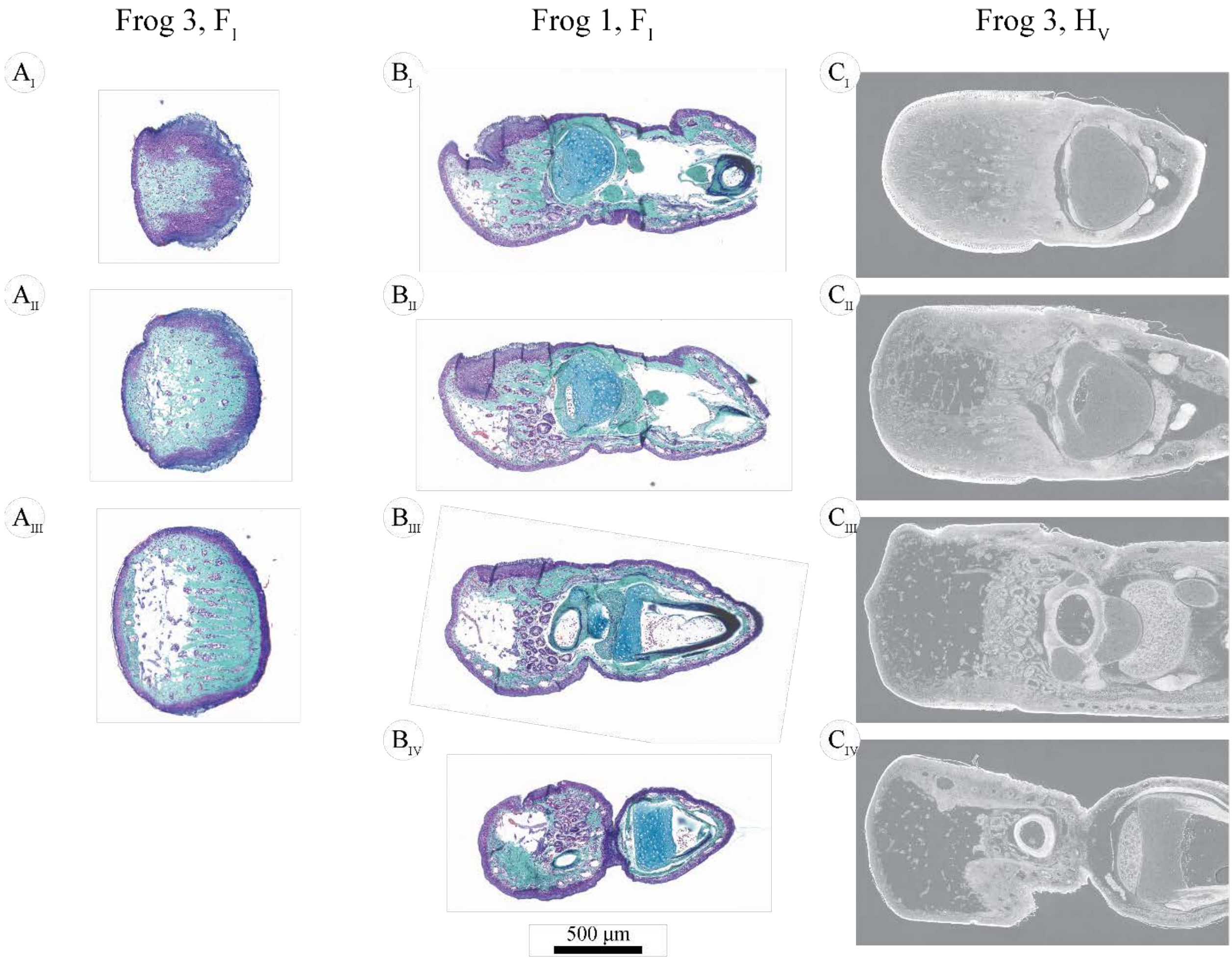

Fig. SI.4. Series of horizontal sections through digital pads of the (A,B) fore- and (C) hindlimbs of *Hyla cinerea* obtained by (A,B) histology and by (C) µ-CT, from ventral to dorsal. The histological sections are stained with Crossmon's light green trichrome including haematoxylin and Alcian blue.

SI.7 Intermediate topologically optimised geometries

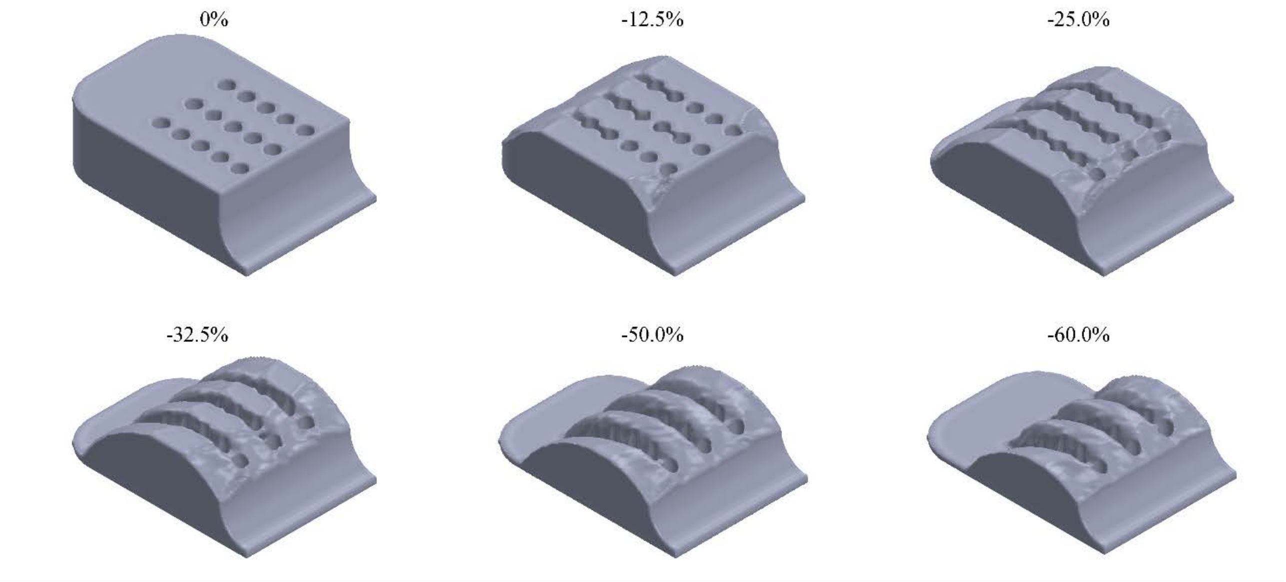

Fig. SI.5. Intermediate geometries resulting from the topological optimisation for increasing volume reduction.

SI.8 Modelling of the normal contact stresses during peeling

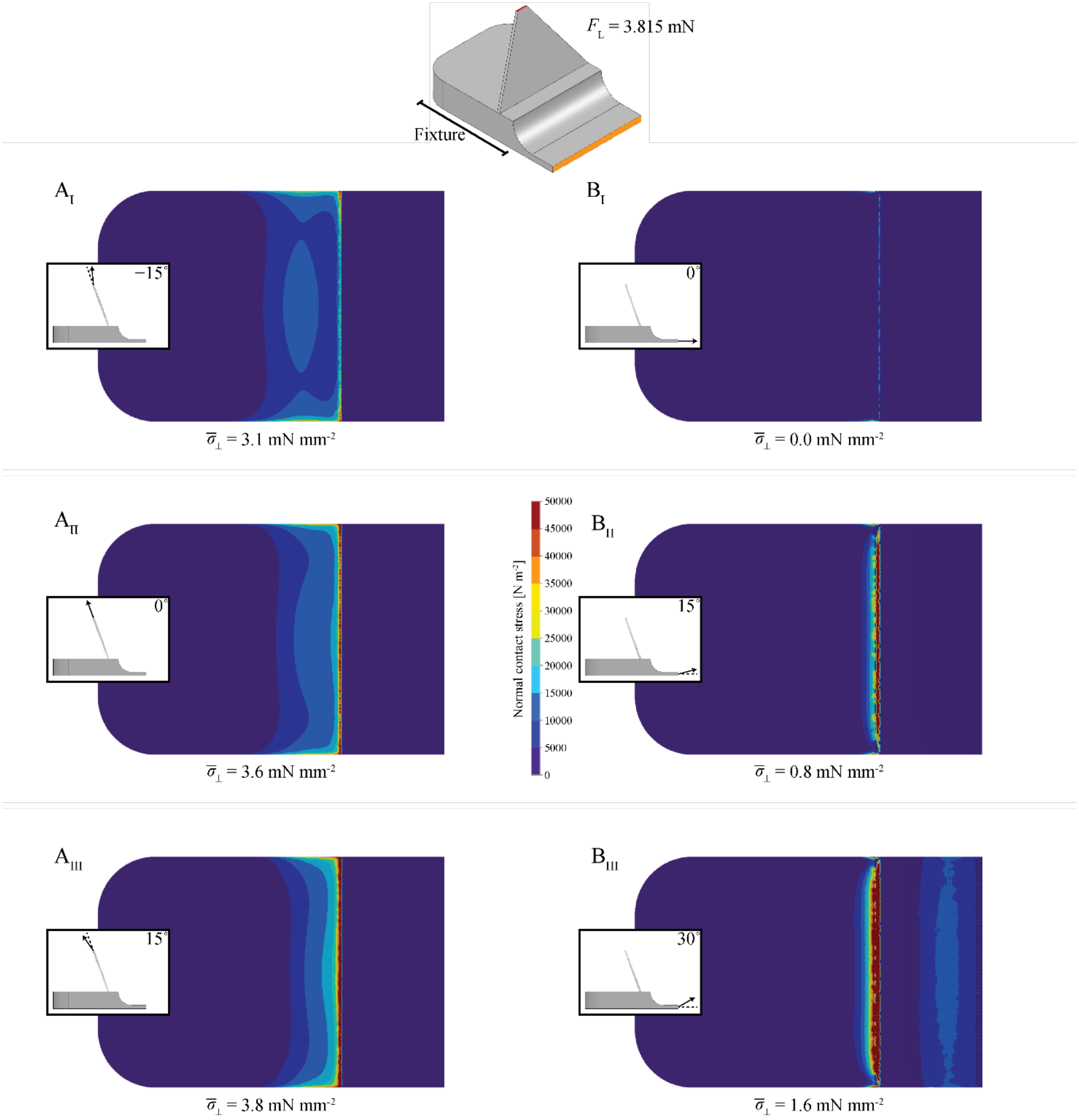

Fig. SI.6. Normal contact stresses in a simplified finite element model (element size = 15 µm) as prediction of the peeling stresses in the digital pads of tree frogs during (A) dorsodistal loading of the septum and (B) proximal loading of the ventral collagen layer. Displacement of the ventral model surface is prohibited as indicated by the black solid line; the proximal model section is not fixed. A load of 3.815 mN is applied on the dorsal surface of the septum (red surface in top model) and on the proximal surface of the ventral collagen layer (orange surface in top model), respectively, at three different angles with respect to the according surface normal. The surface plots show the spatial distribution and the average value of the normal contact stresses at the ventral model surface.
